# Decreased surfactant lipids correlate with lung function in chronic obstructive pulmonary disease (COPD)

**DOI:** 10.1371/journal.pone.0228279

**Published:** 2020-02-06

**Authors:** Christina W. Agudelo, Britta K. Kumley, Estela Area-Gomez, Yimeng Xu, Abdoulaye J. Dabo, Patrick Geraghty, Michael Campos, Robert Foronjy, Itsaso Garcia-Arcos

**Affiliations:** 1 Department of Medicine, SUNY Downstate Medical Center, New York, New York, United States of America; 2 Department of Neurology, Columbia University, New York, New York, United States of America; 3 Department of Cell Biology, SUNY Downstate Medical Center, New York, New York, United States of America; 4 Division of Pulmonary, Allergy, Critical Care and Sleep Medicine, University of Miami Miller School of Medicine, Miami, Florida, United States of America; University of Alberta, CANADA

## Abstract

Smoke exposure is known to decrease total pulmonary surfactant and alter its composition, but the role of surfactant in chronic obstructive pulmonary disease (COPD) remains unknown. We aimed to analyze the compositional changes in the surfactant lipidome in COPD and identify specific lipids associated with pulmonary function decline. Bronchoalveolar lavage (BAL) fluid was obtained from 12 former smokers with COPD and 5 non-smoking, non-asthmatic healthy control volunteers. Lipids were extracted and analyzed by liquid chromatography and mass spectrometry. Pulmonary function data were obtained by spirometry, and correlations of lung function with lipid species were determined. Wild-type C57BL/6 mice were exposed to 6 months of second-hand smoke in a full-body chamber. Surfactant lipids were decreased by 60% in subjects with COPD. All phospholipid classes were dramatically decreased, including ether phospholipids, which have not been studied in pulmonary surfactant. Availability of phospholipid, cholesterol, and sphingomyelin in BAL strongly correlated with pulmonary function and this was attributable to specific lipid species of phosphatidylcholine with surface tension reducing properties, and of phosphatidylglycerol with antimicrobial roles, as well as to other less studied lipid species. Mice exposed to smoke for six months recapitulated surfactant lipidomic changes observed in human subjects with COPD. In summary, we show that the surfactant lipidome is substantially altered in subjects with COPD, and decreased availability of phospholipids correlated with decreased pulmonary function. Further investigation of surfactant alterations in COPD would improve our understanding of its physiopathology and reveal new potential therapeutic targets.

## Introduction

Pulmonary surfactant is a requirement for effective gas exchange and optimal respiratory function. In the alveoli, surfactant lines the luminal surface and it is responsible for decreasing surface tension during inspiration and for preventing alveolar collapse at the end of the respiratory cycle. In the small airways, surfactant maintains airway patency [1, 2]. The study of surfactant has seen a renewed interest in the last decade, partly owing to the availability of new and sensitive techniques like mass spectrometry [3, 4]

Environmental and genetic factors can originate surfactant insufficiency [5]. Premature birth leads to surfactant deficiency in neonates and cause respiratory distress syndrome (RDS) for which exogenous surfactant therapy is routinely administered [6–8]. In adults, surfactant is involved in multiple pulmonary diseases: adult, acute respiratory distress syndrome (ARDS) is associated with surfactant imbalances between lipid species and impaired function [9, 10]; ARDS courses with surfactant deficiency (reviewed in [11]; pneumonia and sepsis-derived acute lung injury are characterized by surfactant deficiency; and cystic fibrosis and idiopathic pulmonary fibrosis is associated with surfactant deficiency and altered lipid composition [12, 13]. In chronic obstructive pulmonary disease (COPD) however, potential alterations in the surfactant lipidome have not been well-characterized. Inhalation of smoke, the most common cause of COPD, is known to impair the properties of surfactant lipids by interfering with its surface tension-lowering properties. Indeed, patients with COPD showed altered lipids in exhaled breath concentrate, a sampling method that collects secretions form upper and lower airways [14]. A clinical study of adults with stable chronic bronchitis showed improvements in pulmonary function after administration of aerosolized phosphatidylcholine (PC) 32:0, the most abundant surfactant phospholipid (PL) [15]. Still, the role of surfactant in the pathogenesis and development of COPD is poorly understood [16–18]

Pulmonary surfactant is synthesized by alveolar type 2 cells (T2C) and it is composed of 90% lipid, primarily phospholipid, with contributions from other lipid classes and ~10% specific surfactant proteins. The surface tension reduction properties of surfactant owe to the predominant lipid, dipalmitoylphosphatidylcholine (PC 32:0) [19]. In addition to surface tension reduction, surfactant lipids also play other roles. Cholesterol allows surfactant recycling by T2C. Phosphatidylglycerol (PG) and phosphatidylinositol (PI) participate in the packaging and extracellular organization of the surfactant complex, as well as in immunomodulation and host defense [20–22].

In this study, we hypothesized that subjects with COPD develop chronic alterations of surfactant composition and homeostasis that persist after smoking cessation. We conducted a systematic lipidomic analysis of bronchoalveolar lavage (BAL) fluid in subjects with COPD that had achieved smoking cessation and compared them with BAL from healthy subjects. COPD patients showed significant alterations in their surfactant lipidomes and exhibited an overall decrease in alveolar total lipid availability that directly correlated with decreased pulmonary function. Forced expiratory volume in one second (FEV1) showed strong correlations with total surfactant lipid, surface tension-lowering PLs, and with novel lipids like bis-monoacyl-phosphoglycerate (BMP) and ether linked-phospholipids, which had not been characterized in pulmonary surfactant. In addition, we show that the commonly used mouse model of second-hand smoke exposure closely recapitulates the lipidomic changes found in human subjects with COPD, thus opening possibilities for future mechanistic research.

## Materials and methods

### Patient population and sample collection

Written consent was obtained from all study participants and approved by the institutional review board of the University of Miami. All consents conformed to the Declaration of Helsinki. BAL and blood were collected from healthy never-smokers and stable COPD patients. Additional COPD BAL samples were obtained from baseline measurements of Feasibility of Retinoids for the Treatment of Emphysema (FORTE) trial participants [23]. In this study, written consent was obtained from all study participants and the trial was approved by the institutional review boards of Columbia University Medical Center, the University of Pittsburgh, the University of California at Los Angeles Medical Center, the University of California at San Diego Medical Center, Boston University Medical Center and Long Island Jewish Medical Center. BAL was performed by instilling 60 ml of saline into the medial or lateral segment of the right middle lobe, with a dwell time of 30 seconds, followed by aspiration. All samples were de-identified prior to analysis.

Demographic characteristics are listed in Table 1. Healthy subjects had no smoking history or asthma, and all COPD patients had achieved self-reported smoke cessation, were stable and had had no exacerbations in the prior 6 months. Subjects were excluded if there was history of systemic corticosteroid use. Patients were receiving standard COPD therapy, including long acting bronchodilators. Specific treatments with long acting beta-agonists (LABA), long acting muscarinic antagonists (LAMA), as well as common comorbidities have been detailed in Table 1 whenever this information was available (for 7 out of 12 patients). BAL levels of pro-inflammatory cytokines are also detailed in Table 1. Pulmonary function was assessed by spirometry and obstruction was calculated by FEV1 as a percent of the predicted value, as per the American Thoracic Society (ATS)/European Respiratory Society (ERS) guidelines [24].

**Table 1:** Patient demographics and inflammatory cytokines.

	Healthy Control	COPD
**N**	5	12
**Age (av. ± S.D)**	64 ± 10	56 ± 8
**Hispanic (%)**	80	50
**Pack Years (av. ± S.D)**	0	35 ± 30*
**BMI (av. ± S.D)**	27 ± 2	29 ± 4
**FEV1/FVC (av. ± S.D)**	0.8 ± 0.08	0.5 ± 0.15**
**% FEV1 (av. ± S.D)**	98.2 ± 0.19	51.7 ± 0.17***
**% FVC (%) (av. ± S.D)**	96.0 ± 0.14	80.5 ± 0.11*
**Plasma TG (mg/dL; av. ± S.D)**	113 ± 44	105 ± 43
**Plasma CE (mg/dL; av. ± S.D)**	129 ± 49	158 ± 35
**IL-6 (pg/ml; av. ± S.D)**	15 ± 10	30 ± 25
**IL-8 (pg/ml: av. ± S.D)**	43 ± 21	41 ± 27
**MCP-1 (pg/ml: av. ± S.D)**	33 ± 41	11 ± 10
**Protein/ELF (ng/ul)**	1.7 ± 1.9	3.0 ± 2.9
**Treatments**		
LABA	0/5	2/7
LAMA	0/5	4/7
**Comorbidities**		
Congestive heart failure, lung edema	0/5	1/7
Cerebrovascular disease, stroke	0/5	1/7

BMI: body mass index; CE: cholesteryl ester; ELF: epithelial lining fluid; FEV1: forced expiratory volume in 1 second; FVC: forced vital capacity; IL: Interleukin; LABA: long acting beta agonists; LAMA: long acting muscarinic antagonists; MCP-1: macrophage chemotactic protein-1; TG: triglyceride.

*p<0.05;

**p<0.005;

***p<0.0005.

### Mouse smoke exposure and BAL

All animal studies were approved by the IACUC at SUNY Downstate Medical Center. Wild-type adult age-matched C57BL/6 mice were exposed to room air or 4-hour of daily second-hand smoke from 20 cigarettes for 6 months using a whole body exposure chamber as in Brehm et al [25]. Briefly, mice were exposed to cigarette smoke from 3R4F reference cigarettes (University of Kentucky, Louisville, KY) in a specially designed chamber (Teague Enterprises, Davis, CA) for a total particulate matter concentration of 80 mg/m^3^. Mice had free access to food and water during the exposures. BAL fluid was obtained by instillation and recovery of 1 mL PBS through a 20G tracheal catheter.

### Analysis of lipids using high performance liquid chromatography-mass spectrometry

Lipid extracts were prepared using a modified Bligh and Dyer method [26], spiked with a cocktail of internal standards, and analyzed using a 6490 Triple Quadrupole LC/MS system (Agilent Technologies, Santa Clara, CA). Glycerophospholipids and sphingolipids were separated with normal-phase HPLC as described before (Chan et al, 2012), with a few modifications. An Agilent Zorbax Rx-Sil column (inner diameter 2.1 x 100 mm) was used under the following conditions: mobile phase A (chloroform:methanol:1 M ammonium hydroxide, 89.9:10:0.1, v/v) and mobile phase B (chloroform:methanol:water:ammonium hydroxide, 55:39.9:5:0.1, v/v); 95% A for 2 min, linear gradient to 30% A over 18 min and held for 3 min, and linear gradient to 95% A over 2 min and held for 6 min. Sterols and glycerolipids were separated with reverse-phase HPLC using an isocratic mobile phase as before [27] except with an Agilent Zorbax Eclipse XDB-C18 column (4.6 x 100 mm). Quantification of lipid species was accomplished using multiple reaction monitoring (MRM) transitions and instrument settings that were developed in earlier studies [27] in conjunction with referencing of appropriate internal standards PA 14:0/14:0, PC 14:0/14:0, PE 14:0/14:0, PG 15:0/15:0, PI 17:0/20:4, PS 14:0/14:0, BMP 14:0/14:0, APG 14:0/14:0, LPC 17:0, LPE 14:0, LPI 13:0, Cer d18:1/17:0, SM d18:1/12:0, dhSM d18:0/12:0, GalCer d18:1/12:0, GluCer d18:1/12:0, LacCer d18:1/12:0, D_7_-cholesterol, CE 17:0, MG 17:0, 4ME 16:0 diether DG, D_5_-TG 16:0/18:0/16:0 (Avanti Polar Lipids, Alabaster, AL). The specific lipid class assigned to each internal standard is detailed in S1 Table. All our results were further normalized to epithelial lining fluid (ELF) for each individual. The dataset for our analysis is available as S2 Table.

### Urea determination and calculation of epithelial lining fluid (ELF)

Lipid concentrations in BAL were normalized to ELF as a surrogate of alveolar surface area, using the method as recommended in Rennard et al [28]. This method is based on the equivalence of urea concentration in ELF and plasma, and it corrects for any dilution of the BAL introduced during collection. Briefly, urea concentration was biochemically measured in plasma and BAL for each patient using a commercially available kit (Biovision, CA), and the dilution factor introduced during the BAL collection and original ELF volume collected was calculated according to the following equation:

$$ELF\;volume=BAL\;volume\;x\;\frac{\left[urea\right]plasma}{\left[urea\right]BAL}$$

No significant differences in the concentration of urea in plasma or BALF were encountered between healthy subjects and COPD patients (S1 Fig). Each subject’s ELF value was used to normalize the corresponding lipid values measured.

### Statistical analysis

All statistical analyses were performed with GraphPad Prism. Average values ± standard error of the mean (SEM) are reported. Identification of outliers was performed with the ROUT method in GraphPad Prism. Values were compared by 2-way ANOVA followed by Bonferroni correction for multiple hypothesis testing. Human subject N was 5 in the healthy group and 12 in the COPD group. Mouse N was 11 in the room air group and 3 in the smoke-exposure group. Significances in the figures are reported as *p<0.05, **p<0.005, ***p<0.0005, and ****p<0.0001.

## Results

### Lipid levels decreased in BAL from COPD subjects

Smoke exposure causes damage to surfactant lipids, but the chronic effects of smoke in the surfactant lipidome in COPD have not been studied. We conducted an untargeted lipidomic analysis in BAL from healthy control and COPD subjects and compared their surfactant lipidomes in relationship with the pulmonary function of each subject.

**Fig 1:**
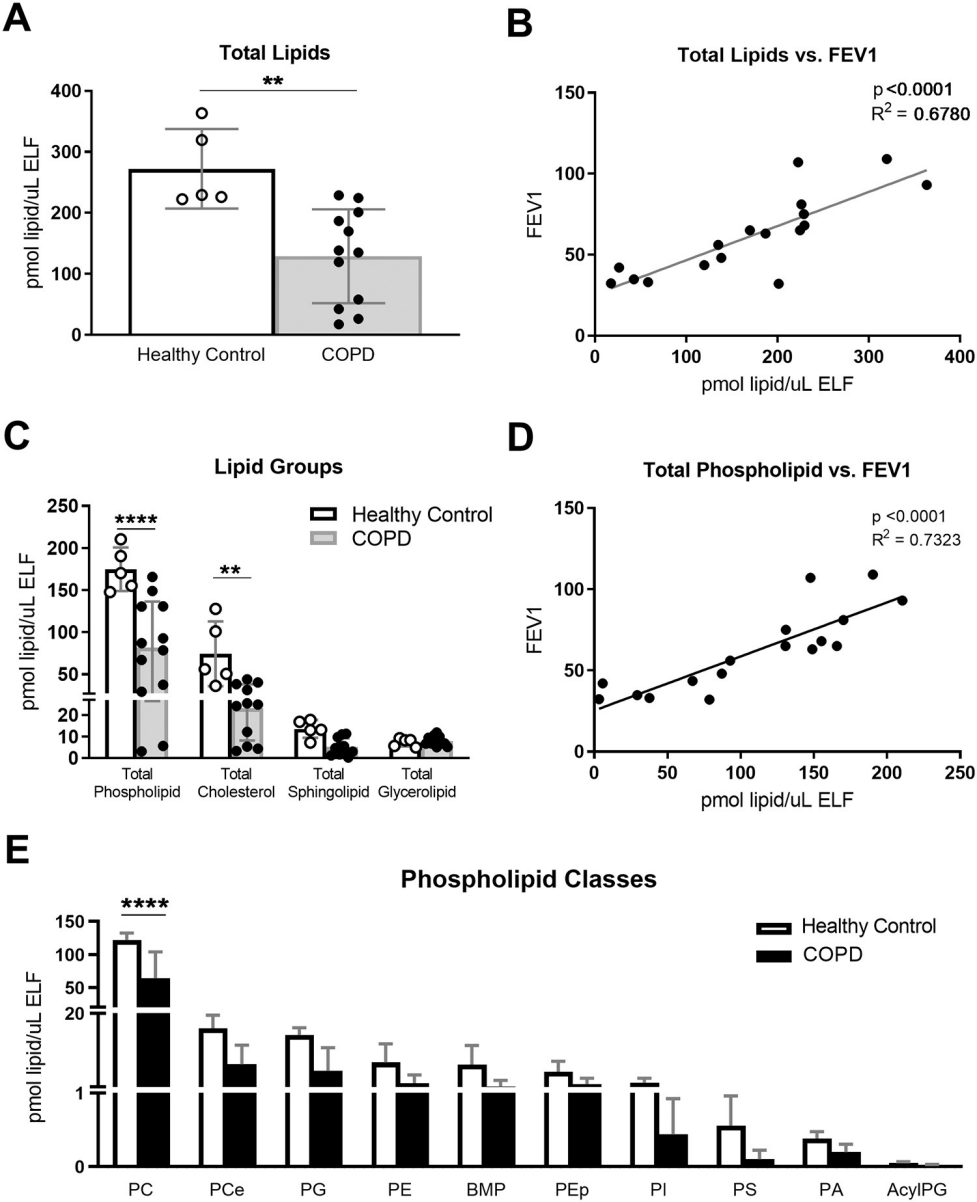
BAL total lipid and lipid classes decreased in COPD correlating with FEV1. BAL was collected from 5 healthy, non-asthmatic control subjects and 12 former smokers with COPD. Lipid concentrations were normalized by ELF. A) Total lipids in BAL, B) total lipids correlated with FEV1, as measured by spirometry, C) lipid quantifications by lipid group, D) total phospholipid correlated with FEV1, and E) lipid quantifications by phospholipid class. Results are reported as average with S.E.M. Linear regressions were performed and p and R2 values are reported. Data was subjected to 2-way-ANOVA statistical analysis with Bonferroni correction for multiple comparisons: *p<0.05, **p<0.005, ***p<0.0005, ****p<0.0001.

The total lipid concentration in BAL from control subjects was 272.2± 29.1 pmol/μl ELF, and it decreased to 129.1 ± 22.2 pmol/μl ELF in subjects with COPD (p<0.005; Fig 1A). The BAL total lipid concentration showed a significant and direct correlation with pulmonary function measured as FEV1 (Fig 1B; p<0.0001, R^2^ = 0.68).

As expected, the most abundant lipid classes detected in BAL were phospholipids and cholesterol, followed by sphingolipids and glycerolipids (Fig 1C). Total phospholipid amounts decreased from 167.9 ± 9.4 pmol/μl ELF in healthy subjects to 81.5 ± 15.9 pmol/μl ELF in subjects with COPD (p<0.001; Fig 1C and S2 Table), and the concentration of total phospholipid correlated strongly with FEV1 (R^2^ = 0.73; p<0.0001; Fig 1D). The phospholipid classes detected are detailed in Fig 1E. For some of these species, like plasmalogen phosphatidylethanolamine (PEp) and BMP, the functions in pulmonary surfactant are unknown. All phospholipid classes showed consistent decreases in COPD subjects.

These data show that COPD is associated with decreased concentration of BAL lipids and that pulmonary function in COPD patients strongly correlates with the availability of alveolar lipid, especially of PL. Findings in the different lipid classes are more exhaustively described below, grouped by their role in the extracellular pulmonary surfactant mixture.

### Surface tension-reducing lipids

**Fig 2:**
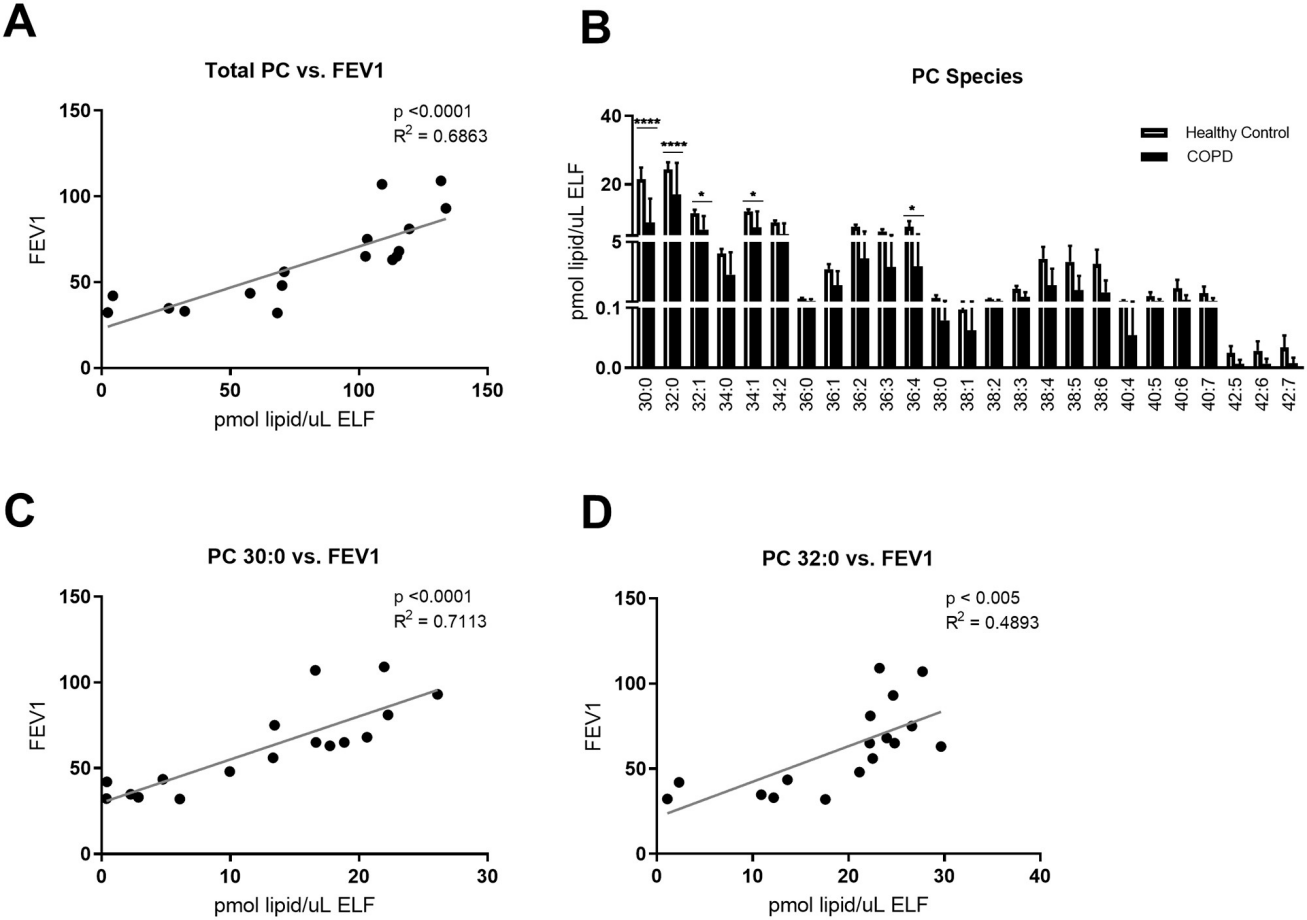
BAL phosphatidylcholine (PC) species decreased in COPD correlating with FEV1. BAL was collected as described, and lipid concentrations were normalized by ELF. A) Total PC concentration correlated with FEV1, as measured by spirometry, B) lipid concentration by PC species, C) PC 30:0 concentration correlated with FEV1, and D) PC 32:0 concentration correlated with FEV1. Linear regressions were performed and p and R^2^ values are reported. Data was subjected to 2-way-ANOVA statistical analysis with Bonferroni correction for multiple comparisons: *p<0.05, **p<0.005, ***p<0.0005, ****p<0.0001.

Amongst the surfactant phospholipids, PC, and specifically PC 32:0, is the most important PL for surface tension reduction. Indeed, FEV1 showed a significant correlation with total concentration of PC (p<0.0001; R^2^ = 0.69, Fig 2A). The most abundant PC species were PC 30:0 and PC 32:0, and both of them showed significant correlations with FEV1 (p<0.0001; R^2^ = 0.71 for PC 30:0; Fig 2C; and p <0.005; R^2^ = 0.49 for PC 32:0; Fig 2D). All PC species showed trending or significant decreases in BALF concentration from COPD subjects (Fig 2B and S2 Table).

These data show a strong correlation of pulmonary function in COPD with lipids with surface tension reducing properties, suggesting increased surface tension may be a contributor to COPD pathophysiology.

### Lipids with roles in surfactant extracellular organization and immunity

**Fig 3:**
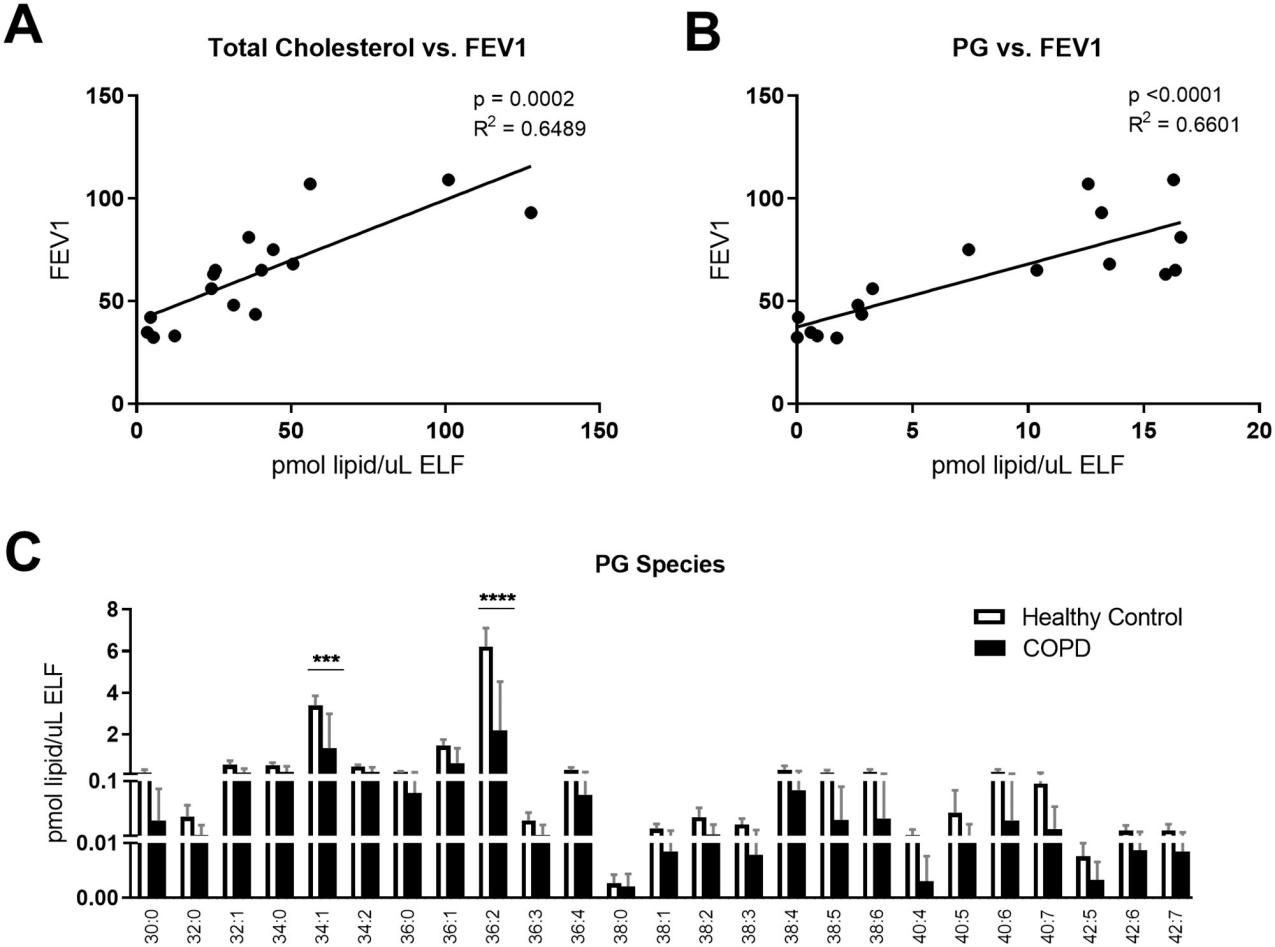
BAL cholesterol and phosphoglycerol (PG) decreased in COPD correlating with FEV1. BAL was collected as described, and lipid concentrations were normalized by ELF. A) Total cholesterol correlated with FEV1, as measured by spirometry, B) PG concentration correlated with FEV1, and C) lipid concentrations by PG species. Linear regressions were performed and p and R2 values are reported. Data was subjected to 2-way-ANOVA statistical analysis with Bonferroni correction for multiple comparisons: *p<0.05, **p<0.005, ***p<0.0005, ****p<0.0001.

Other lipids than PL are required in surfactant for appropriate dynamics of the surfactant film in the extracellular lining. Cholesterol aids in the surfactant film extracellular spreading and recycling processes. Cholesterol was the second most abundant lipid group detected, comprising 25% of the BAL lipid in healthy subjects. In COPD patients, cholesterol in BAL was decreased by ~40%, from 74.4 ± 17.2 pmol/μl ELF in healthy controls, to 23.1 ± 4.5 pmol/μl ELF (p<0.005; Fig 1C). The concentration of cholesterol in BAL also correlated positively with FEV1 (p<0.0005, R^2^ = 0.65; Fig 3A).

PG partakes in the extracellular organization of the surfactant film and also in innate immune processes [20–22]. The concentration of PG in BAL correlated with FEV1 (p<0.0005, R^2^ = 0.66; Fig 3B), and the most abundant PG species, PG 36:2, significantly decreased in the diseased state, with COPD subjects having ~ 30% of the PG of healthy controls (p<0.0001; Fig 3C). Other less abundant species also showed trending or significant decreases (S2 Table).

Individuals with COPD often show increased abundance of pro-inflammatory lipids and mediators in exhaled breath condensate [14]. In our cohort, ceramide concentration in BAL was not significantly altered by COPD, nor did it meet significant correlation with FEV1 (p = 0.053, R^2^ = 0.24; S2 Table). Consistently, BAL levels of pro-inflammatory cytokines IL-6, IL-8 and MCP-1 did not differ between healthy and COPD subjects (Table 1).

Together, these data show COPD-associated deficiencies in surfactant lipids previously described to impact the fluid dynamics of the film in the alveolar space and also its immune function.

### Newly identified and surfactant lipids of unknown function

**Fig 4:**
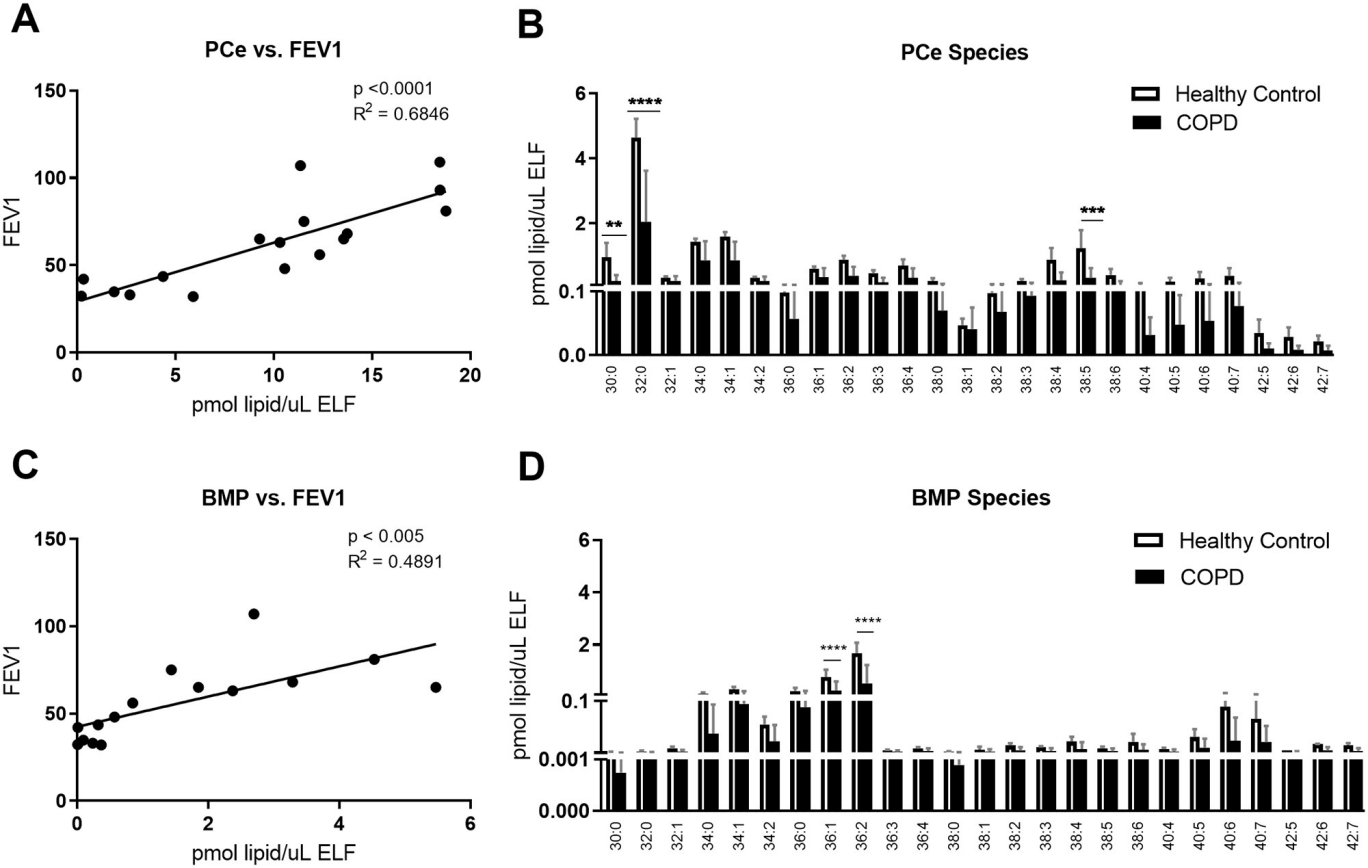
Novel surfactant lipids decreased in COPD correlating with FEV1. BAL was collected as described, and lipid concentrations were normalized by ELF. A) Phosphatidylcholine ester (PCe) concentration correlated with FEV1, as measured by spirometry, B) lipid concentration by PCe species, C) bis(monoacylglycero)phosphate (BMP) concentration correlated with FEV1, and D) lipid concentrations by BMP species. Linear regressions were performed and p and R2 values are reported. Data was subjected to 2-way-ANOVA statistical analysis with Bonferroni correction for multiple comparisons: *p<0.05, **p<0.005, ***p<0.0005, ****p<0.0001.

Our approach allowed for the identification of lipids that have not been thoroughly described in pulmonary surfactant previously. These lipids include ether-linked PLs and BMPs. Ether PC (PCe) was the second major phospholipid identified in the BAL, representing 10% of total PL. Ether phospholipids have the same chemical structure as PLs, with the exception that the fatty acid in the sn-1 position is linked by an ether instead of an ester bond. The concentration of total PCe correlated with FEV1 (p<0.0001, R^2^ = 0.68; Fig 4A). Similar to ester PC, PCe with the acyl composition 32:0 was the most abundant, with other PCe containing short and saturated acyl chains also being highly represented. PCe 30:0, PCe 32:0, and PCe 38:5, showed significant decreases in surfactant from COPD patients (p<0.005 for PC 30:0, p<0.0001 for 32:0, and p<0.0005 for 38:5; Fig 4B).

BMP, also known as lysobisphosphatidic acid, is a marker of late endosomal membranes [29]. Thus far, BMP does not have a described function in alveolar surfactant metabolism. In our cohort, BMP 36:1 and 36:2 were the most abundant species detected, and a significant decrease was observed for both in COPD subjects (p<0.0001; Fig 4D), as well as trends for other species and total BMP levels (S2 Table). BAL concentration of BMP strongly correlated with FEV1 (p<0.005, R^2^ = 0.49, Fig 4C).

**Fig 5:**
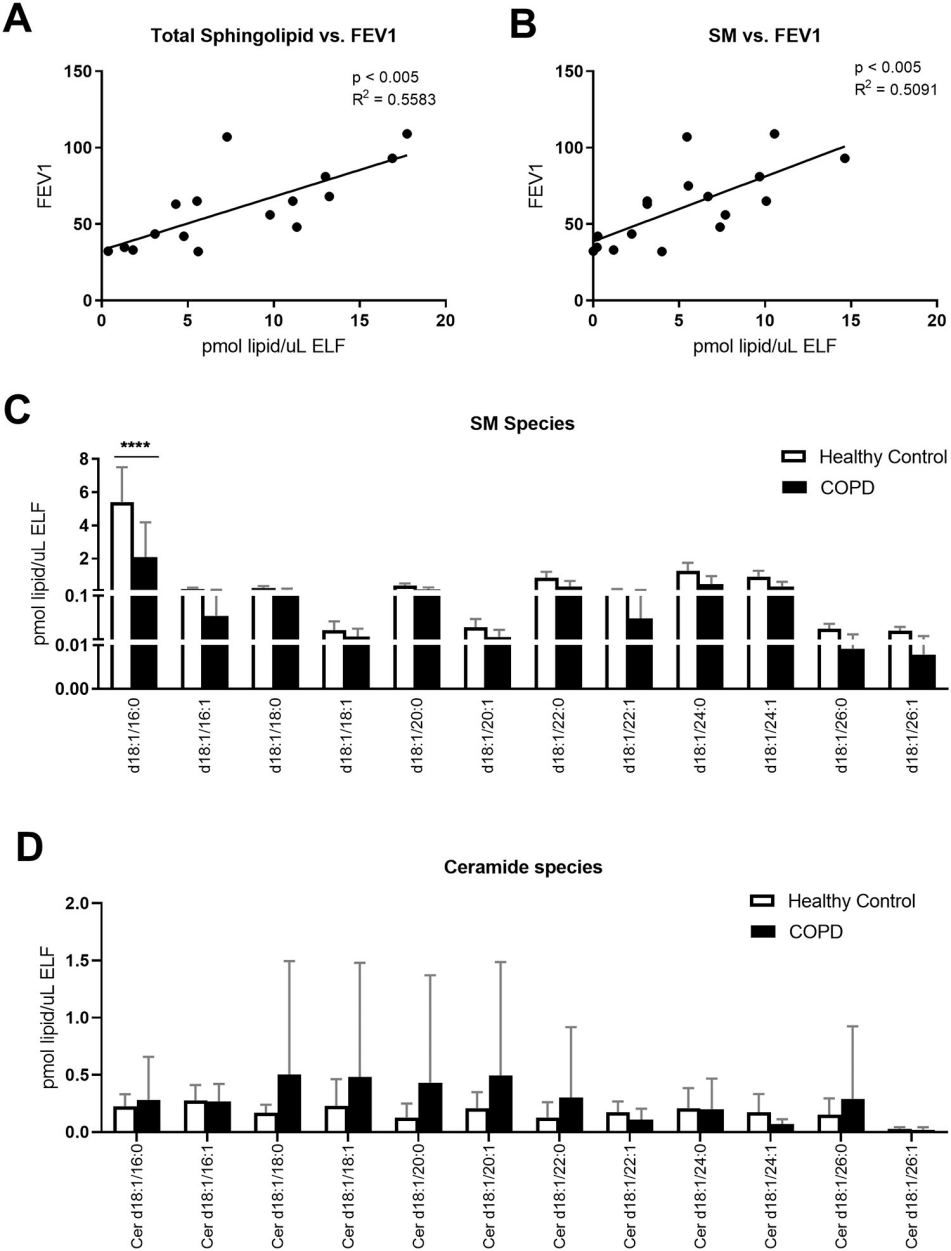
BAL sphingolipid (SL) and sphingomyelin (SM) decreased in COPD correlating with FEV1. BAL was collected as described, and lipid concentrations were normalized by ELF. A) Total SL concentration correlated with FEV1, as measured by spirometry, B) SM concentration correlated with FEV1, C) lipid concentrations by SM species and D) lipid concentrations by ceramide species. Linear regressions were performed and p and R2 values are reported. Data was subjected to 2-way-ANOVA statistical analysis with Bonferroni correction for multiple comparisons: *p<0.05, **p<0.005, ***p<0.0005, ****p<0.0001.

We detected other lipids that have been previously described in surfactant, but their functions are still unclear. Sphingolipids (SL), for instance, were highly abundant. Total SL concentration showed a correlation with FEV1 (p<0.005; R^2^ = 0.07; Fig 5A) and this was attributable to the most abundant SL, sphingomyelin (SM; p <0.005; R^2^ = 0.51; Fig 5B). All species of SM were decreased in COPD patients, especially the most predominant SM, d18:1/16:0 (p<0.0001; Fig 5C and S2 Table). Ceramides, also sphingolipids, were found at low abundance and with high variation between different individuals (Fig 5D). No statistically significant differences were found in ceramide species between the healthy control and the COPD groups.

Amongst the less abundant PLs, phosphatidylinositol (PI), phosphatidic acid (PA) and phosphatidylserine (PS) of nearly all acyl compositions were decreased in COPD subjects, and the most abundant species showed statistical significance. (S2 Table).

### Lipids able to disrupt surfactant’s functions

Some lipid classes are normally maintained at a minimum in the alveolar surfactant mixture because they could otherwise interfere with its normal functions. Lysophospholipids (LPLs) in the alveoli can arise as degradation products of PL, and an increase in LPL concomitant with a decrease in PL could indicate enhanced alveolar PL degradation. In this cohort, there was no significant increase in LPL in the BAL of COPD subjects (S2 Fig). LPL showed low abundance, and while there were some trends toward increased ratios of lysophospholipid to phospholipid, such as LPE /PE or LPS/PS, none of them reached significance (S2 Fig).

Triacylglycerols (TG) and cholesteryl esters (CE) are apolar lipids normally kept in low concentrations in pulmonary surfactant. There was a decreasing trend in the concentration of all CE species in subjects with COPD (S2 Table). In this cohort, the most abundant CE in BAL was CE 18:2 (S2 Table), which is also the most abundant plasma CE species in humans. To discard potential leakage from the plasma into the alveolar space, we measured plasma lipids. There was no significant correlation between plasma and BAL CE levels (S3A Fig). Similarly, plasma TG levels did not correlate with surfactant TG levels (S3B Fig).

The data above suggest that defective permeability barrier function between plasma and the alveolar compartment is unlikely to cause the reductions in surfactant PL in COPD patients.

### Second-hand smoke exposure in mice recapitulated human COPD findings

The findings in our human cohort suggest that surfactant could be a new focus of study in the pathophysiology of COPD. We performed a pilot study asking if an animal model of COPD would recapitulate the surfactant lipidomic changes associated with human COPD and make a useful model for future advancement in this field. Exposure of mice to second-hand smoke is a commonly used model for the study of human COPD. Wild-type C57BL/6 mice were exposed to room air or to six-months of second-hand smoke, a period of time and modality of smoke exposure that is known to cause decline in human pulmonary function [24].

**Fig 6:**
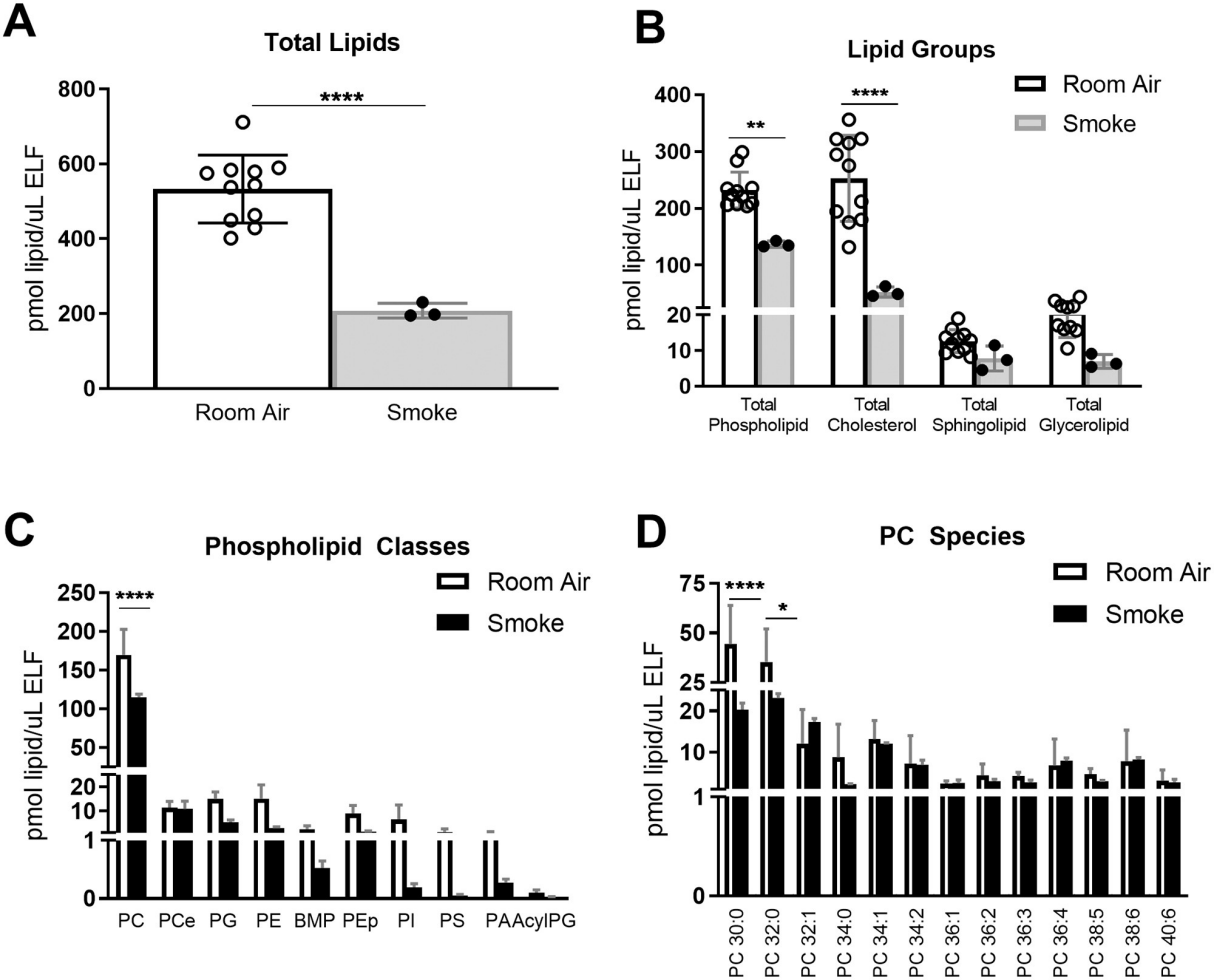
Mouse COPD model recapitulated surfactant alterations in human COPD subjects. BAL was collected from 14 age-matched WT mice, 11 exposed to 6 months of room air and 3 exposed to 6 months of second-hand smoke. BAL lipid concentrations were normalized by ELF. A) Total lipid concentrations, B) lipid concentrations by lipid group, C) by phospholipid, and D) by PC species. Data was subjected to 2-way-ANOVA statistical analysis with Bonferroni correction for multiple comparisons: *p<0.05, **p<0.005, ***p<0.0005, ****p<0.0001.

Lipidomic analysis of BAL from smoke-exposed mice showed compositional changes similar to those observed in human COPD subjects. Total lipid amount decreased from 532.93 ± 27.3 pmol/μl ELF in room-air exposed mice to 207.9 ± 11.4 pmol/μl ELF in smoke-exposed mice (p<0.0001, Fig 6A). Phospholipid and cholesterol were the most abundant lipid classes in mice, and they were both significantly decreased by smoke exposures (p<0.005 for PL and p<0.0001 for cholesterol; Fig 6B). As expected, PC was the most abundant PL class, and short and saturated PCs were the most abundant species (Fig 6C). PC 30:0 and PC32:0 showed a significant decrease in smoke-exposed mice, and this caused a statistically significant decrease in total amounts of PC (p<0.0001; Fig 6). Other PL classes also showed reductions in BAL from smoke-exposed mice (Fig 6C). The main difference between human COPD and mouse smoke-exposures was the less prominent role and lack of change in PCe in mice, as well as a partial compensation by PC32:1 in mice that did not occur in human subjects. However, PC30:0 and PC 32:0 significantly decreased in smoke-exposed mice and other abundant PC species showed consistent trends. Overall, this mouse model faithfully replicated the surfactant lipid decreases and alterations discovered for human COPD subjects.

## Discussion

Smoke exposure exerts harmful effects on the metabolism of extracellular surfactant and in surfactant-producing alveolar type 2 cells [5, 17]. However, the involvement of surfactant lipids in the pathogenesis and development of COPD has not been studied in depth. To our knowledge, this is the first study examining the composition of surfactant collected directly from the lower airways and alveoli of former smokers with stable COPD and defining relationships between specific lipids and FEV1. Our study shows that COPD subjects have decreased availability of total surfactant lipid, and specifically the concentrations of PL and FC are dramatically decreased. Simultaneously, BAL concentration of these and other less studied lipids significantly correlate with pulmonary function.

The clinical umbrella classification of COPD includes varying degrees of airway inflammatory remodeling and emphysematous destruction of alveolar tissue. Loss or dysfunction of type 2 cells subsequent to chronic smoke exposure could offer an explanation for the decreased surfactant lipid availability in COPD patients. The fact that all classes of PL were decreased despite originating from different synthetic pathways [30] suggests a secretion defect in COPD, rather than a synthetic one. This idea is supported by the decrease of BMP amongst the BAL lipids. BMP is specific of lysosomal inner membranes and its presence in BAL could originate from the exocytosis of lamellar bodies, which are lysosome-related organelles. Alternatively, enhanced extracellular PLA_2_ activity and subsequent catabolism of PL to LPL, which is associated with inflammation, could further explain the decreased surfactant PL in COPD patients [31, 32]. We did not find significantly increased LPL/PL ratios in COPD (S2 Fig), but LPL have a short life and do not accumulate, challenging their accurate detection.

We detected relevant amounts of PCe and PEp in pulmonary surfactant, and PCe showed decreased availability and significant correlation with FEV1. The biophysical properties of ether-linked PLs are somewhat different from those of the more studied ester-linked PLs. The role of PCe species in surfactant has not been addressed and it remains a question if PCe32:0 exhibits surface tension-reduction properties similar to those of PC32:0.

Lipid quantitative data were normalized to ELF for a variety of reasons. Since surfactant covers the inner surface of alveoli and small airways, ELF volume was used as a surrogate for the alveolar area. The amount of ELF is proportional to the internal lung area that needs to be covered by the surfactant mixture [33]. BAL total protein or albumin concentration were discarded as potential normalizers because they can be altered in disease. Also, the ATS/ERS guidelines discourage from reporting lipid amounts relative to BAL volume to prevent inaccuracies related with the lack of standardization for BAL volumes during the procedure [34]. Therefore, normalizing the lipidomic data by ELF was considered the most meaningful manner of expressing surfactant data, and in this case it also accounts for the expected decrease in alveolar surface area in COPD subjects.

Besides reducing surface alveolar tension, surfactant lipids partake in other processes, like immune defense. PG and PI have antimicrobial properties [21, 22] and their decreased availability suggests yet another possible mechanism for the higher risk of infections during COPD that often results in disease exacerbations. The study by Telenga et al [14] showed increased concentrations of inflammatory marker ceramide in smokers with COPD but not after smoking cessation. Consistently, we did not detect significant alterations in extracellular ceramides in our population of COPD patients that had achieved smoke cessation.

Patients with stable chronic bronchitis showed improved FEV1 and other measures of lung function after administration of exogenous surfactant, which points at a potential therapeutic value for surfactant targeting despite COPD is not caused by surfactant deficiency [15]. It was recently reported that up to 50% of COPD patients develop concomitant fibrosis [35], raising the question of whether decreasing surface tension in alveoli and small airways and preventing collapse could provide a benefit for these patients.

Our analysis opens multiple scientific questions to be further pursued for better understanding of the roles and potential therapeutic targeting of surfactant lipids in COPD. We found that a commonly used research model, C57BL/6 mice exposed to second-hand smoke for a 6-month period, closely replicates the surfactant lipid phenotype of human COPD subjects. The surfactant lipidomes of humans and mice share a high degree of similarity, with minor differences. Disaturated PC accounts for approximately 67% of the surfactant composition in humans, and for approximately 45% in mice [36]. Studies performed using different methods of lipid extraction and analysis could report different proportional estimates of surfactant components [37]. In our pilot cohort of mice, changes in their surfactant lipidome mirrored those of humans, so we consider the mouse model to be useful for further pre-clinical studies.

**Fig 7:**
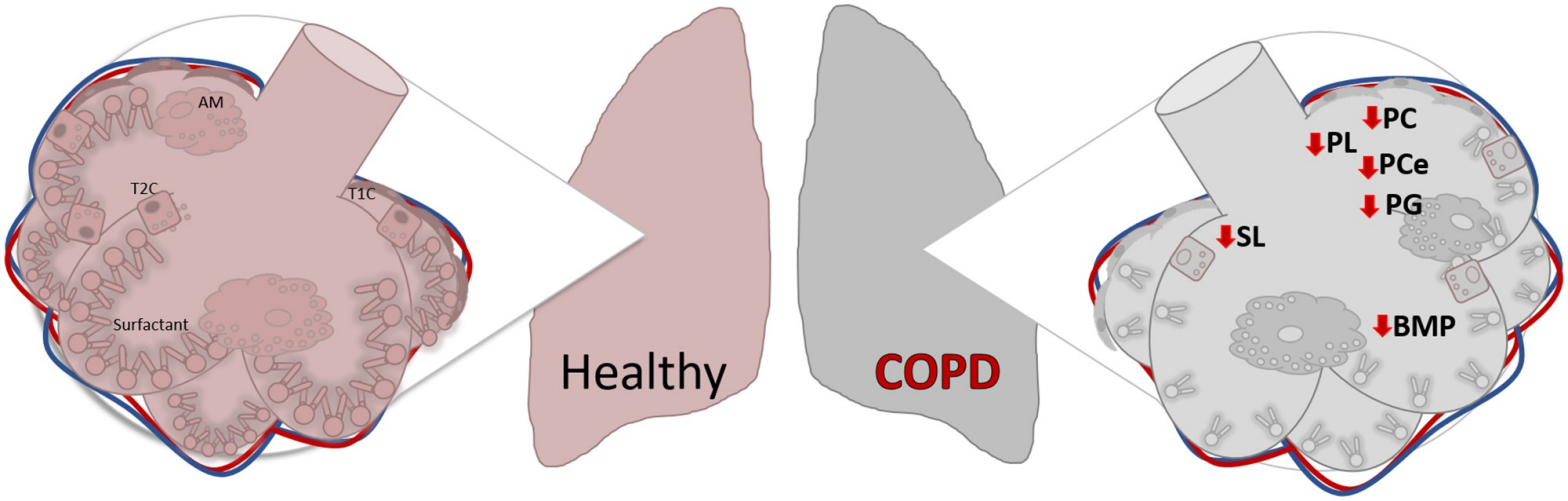
Diagram representing major lipid changes in BAL during COPD. Abbreviations: T1C: type 1 cell; T2C: type 2 cell; AM: alveolar macrophage; BMP: Bis(monoacylglycero)phosphate; PL: phospholipid; PC: phosphatidylcholine; PCe: ether phosphatidylcholine; SL: sphingolipids; SM: sphingomyelin.

In summary, this study describes for the first time that individuals with COPD have surfactant lipidome permanent and significantly alterations that correlate with disease severity (Fig 7). It also uncovers new players in the pathology of COPD that may be further studied to elucidate implications of surfactant homeostasis and its potential for therapeutic targeting.

## Supporting information

S1 Fig Urea content of human and mouse BAL and plasma. BAL was collected from 5 healthy, non-asthmatic control subjects and 12 former smokers with COPD, and from 14 age-matched WT mice, 11 exposed to 6 months of room air and 3 exposed to 6 months of second-hand smoke. Lipid concentrations were normalized by ELF. Urea measurements from A) human BAL, B) human plasma, C) mouse BAL, and D) mouse plasma.
(TIF)

S2 Fig BAL lysophospholipid/phospholipid comparisons. BAL was collected as described, and lipid concentrations were normalized by ELF. The ratios of specific lysophopholipid to phospholipid species are depicted in the following order: A) LPC/PC, B) LPCe/PCe, C) LPE/PE, D) LPEp/PEp, E) LPI/PI, and F) LPS/PS. Data was subjected to 2-way-ANOVA statistical analysis with Bonferroni correction for multiple comparisons: *p<0.05, **p<0.005, ***p<0.0005, ****p<0.0001.
(TIF)

S3 Fig BAL and plasma cholesterol and triglyceride (TG) comparisons. BAL was collected as described, and lipid concentrations were normalized by ELF. Correlation of BAL and plasma A) cholesterol and B) TG. Linear regressions were performed and p and R2 values are reported.
(TIF)

S1 Table Internal standards used for each lipid class in the analysis of lipids by HPLC/MS. (DOCX)

S2 Table Concentration of lipids detected in BAL from healthy and COPD subjects. (DOCX)

## Abbreviations

AC: acyl carnitine
AcylPG: acyl phosphatidylglycerol
BAL: bronchoalveolar lavage
BMP: Bis(monoacylglycero)phosphate
CE: cholesterol ester
Cer: ceramide
COPD: chronic obstructive pulmonary disease
DG: diacylglycerol
ELF: epithelial lining fluid
FC: free cholesterol
FEV1: forced expiratory volume in 1 second
FVC: forced vital capacity
LPC: lysophosphatidylcholine
LPCe: ether lysophosphatidylcholine
LPE: lysophosphatidylethanolamine
LPEp: plasmalogen lysophosphatidylethanolamine
LPI: lysophosphatidylinositol
LPL: lysophospholipid
LPS: lysophosphatidylserine
MG: monoacylglycerol
PA: phosphatidic acid
PC: phosphatidylcholine
PCe: ether phosphatidylcholine
PE: phosphatidylethanolamine
PEp: plasmalogen phosphatidylethanolamine
PG: phosphatidylglycerol
PI: phosphatidylinositol
PL: phospholipid
PS: phosphatidylserine
RDS: respiratory distress syndrome
SM: sphingomyelin
TG: triacylglycerol
