# Supplementary figures and images for: Decreased surfactant lipids correlate with lung function in chronic obstructive pulmonary disease (COPD)
Source: PLoS One. 2020 Feb 6;15(2):e0228279. doi: 10.1371/journal.pone.0228279 (PMC7004328; doi:10.1371/journal.pone.0228279)

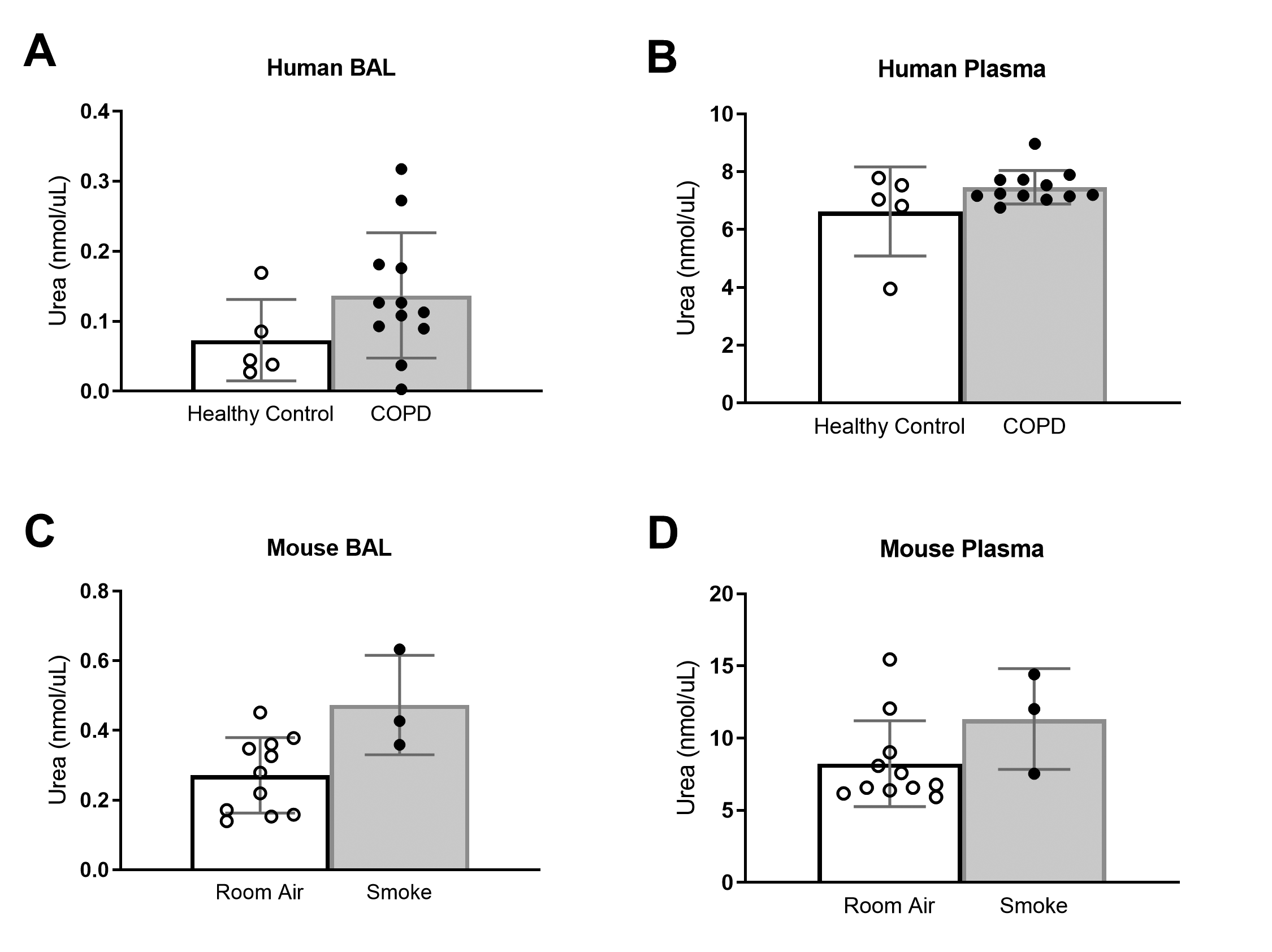

Supplement: S1 Fig — BAL was collected from 5 healthy, non-asthmatic control subjects and 12 former smokers with COPD, and from 14 age-matched WT mice, 11 exposed to 6 months of room air and 3 exposed to 6 months of second-hand smoke. Lipid concentrations were normalized by ELF. Urea measurements from A) human BAL, B) human plasma, C) mouse BAL, and D) mouse plasma. (TIF) [file pone.0228279.s001.tif]

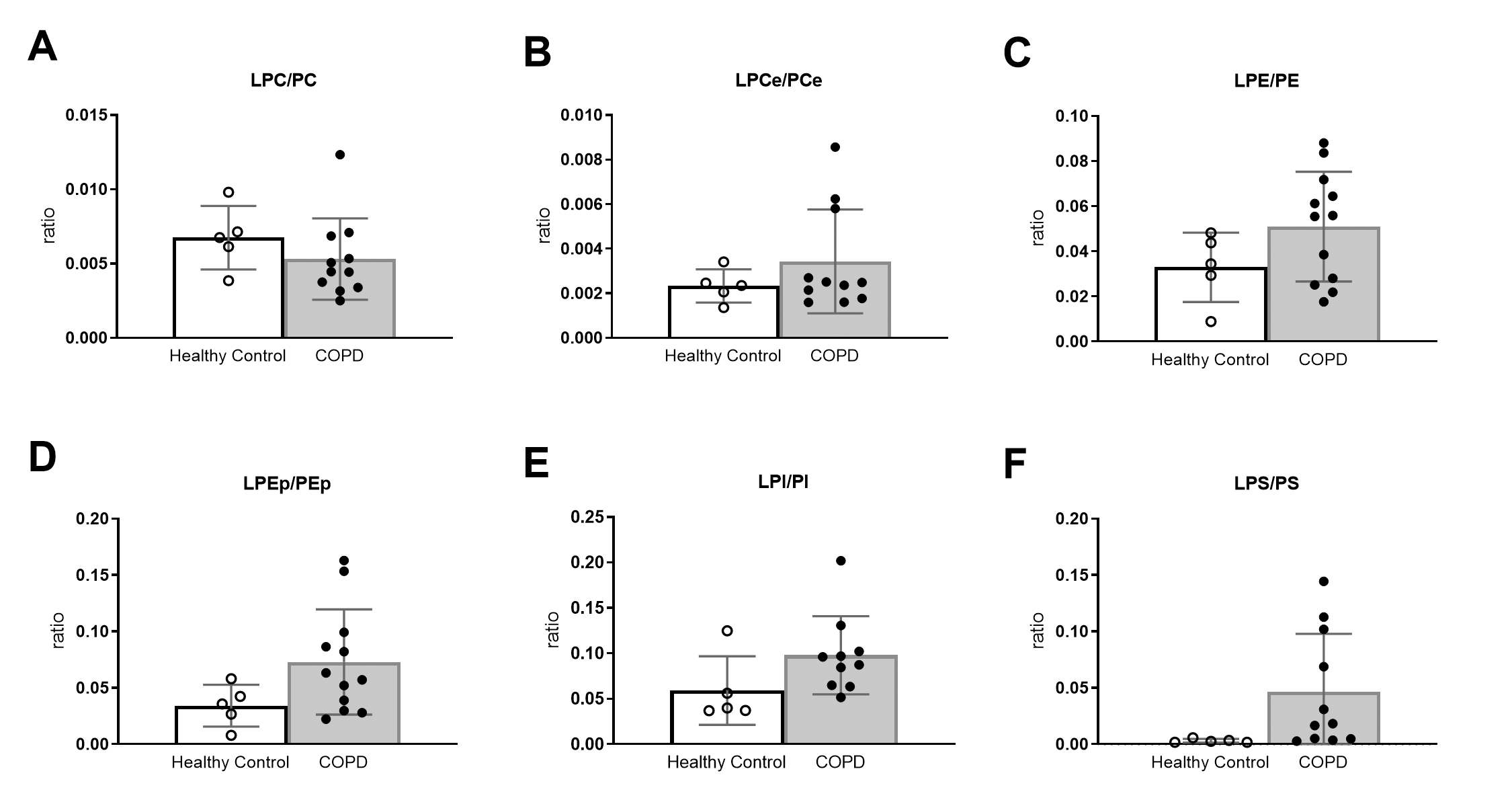

Supplement: S2 Fig — BAL was collected as described, and lipid concentrations were normalized by ELF. The ratios of specific lysophopholipid to phospholipid species are depicted in the following order: A) LPC/PC, B) LPCe/PCe, C) LPE/PE, D) LPEp/PEp, E) LPI/PI, and F) LPS/PS. Data was subjected to 2-way-ANOVA statistical analysis with Bonferroni correction for multiple comparisons: *p<0.05, **p<0.005, ***p<0.0005, ****p<0.0001. (TIF) [file pone.0228279.s002.tif]
