# Supplementary material for: Decreased surfactant lipids correlate with lung function in chronic obstructive pulmonary disease (COPD)
Source: PLoS One. 2020 Feb 6;15(2):e0228279. doi: 10.1371/journal.pone.0228279 (PMC7004328; doi:10.1371/journal.pone.0228279)
Supplement: S1 Table — (DOCX) [file pone.0228279.s004.docx]

| Internal Standards (IS) | Classes using IS |
| --- | --- |
| IS Chol d7 | FC |
| IS CE C17 | CE |
| IS MAG C17 | MG |
| IS DG 28:0/14:0 | DG |
| IS TG 50:0 d5 | TG |
| IS Cer C17:0 | Cer, dhCer |
| IS SM d18:1/12:0 | SM |
| IS dhSM d18:0/12:0 | dhSM |
| IS MHCer d18:1/12:0 | MHCer |
| IS Sulf d18:1/12:0 | Sulf |
| IS LacCer d18:1/12:0 | GB3, LacCer |
| IS PA 28:0 | PA |
| IS PC 28:0 | PC, PCe |
| IS PE 28:0 | PE, PEp |
| IS PG 15:0/15:0 | PG |
| IS PI 37:4 | GM3, PI |
| IS PS 28:0 | NSer, LPS, PS |
| IS LPC 13:0 | LPC, LPCe |
| IS LPE 14:0 | LPE, LPEp |
| IS LPI 13:0 | LPI |
| IS BMP 28:0 | BMP |
| IS AcylPG 14:0 | AcylPG, NAPE, NAPS |

**S1 Table. Internal standards used for each lipid class in the analysis of lipids by HPLC/MS.**
