# Supplementary material for: Decreased surfactant lipids correlate with lung function in chronic obstructive pulmonary disease (COPD)
Source: PLoS One. 2020 Feb 6;15(2):e0228279. doi: 10.1371/journal.pone.0228279 (PMC7004328; doi:10.1371/journal.pone.0228279)
Supplement: S2 Table — (DOCX) [file pone.0228279.s005.docx]

| **S2 Table. Concentration of lipids detected in BAL from healthy and COPD subjects.** | | | | | |
| --- | --- | --- | --- | --- | --- |
|  |  |  |  |  |  |
| **LIPID GROUPS** | | | | | |
|  | **Average Lipid (pmol/µL ELF)** | | **Standard Error** | |  |
| **Lipid** | **Healthy Control** | **COPD** | **Healthy Control** | **COPD** | p value |
| **Total Phospholipids** | 170.069 | 81.511 | 8.744 | 15.902 | **<0.0001** |
| **Total Lysophospholipids** | 1.230 | 0.627 | 0.124 | 0.141 | >0.9999 |
| **Total Sphingolipids** | 13.633 | 8.015 | 1.850 | 2.851 | >0.9999 |
| **Total Neutral Lipids** | 5.181 | 1.452 | 1.569 | 0.396 | >0.9999 |
| **Total Lipids** | 267.501 | 117.613 | 26.142 | 21.227 | **<0.0001** |
| **LIPID CLASSES** | | | | | |
|  | **Average Lipid (pmol/µL ELF)** | | **Standard Error** | |  |
| **Lipid** | **Healthy Control** | **COPD** | **Healthy Control** | **COPD** | p value |
| **FC** | 69.361 | 19.201 | 16.168 | 4.502 | **<0.0001** |
| **PC** | 121.929 | 63.806 | 4.763 | 13.461 | **<0.0001** |
| **PCe** | 16.144 | 6.905 | 1.519 | 1.634 | 0.091 |
| **PE** | 7.354 | 2.020 | 2.158 | 0.711 | >0.9999 |
| **PEp** | 4.977 | 1.724 | 1.181 | 0.523 | >0.9999 |
| **PS** | 0.556 | 0.101 | 0.181 | 0.041 | >0.9999 |
| **PI** | 2.139 | 0.438 | 0.513 | 0.162 | >0.9999 |
| **PG** | 14.433 | 5.175 | 0.834 | 1.992 | 0.088 |
| **PA** | 0.385 | 0.197 | 0.040 | 0.036 | >0.9999 |
| **BMP** | 3.504 | 1.130 | 0.419 | 0.523 | >0.9999 |
| **AcylPG** | 0.050 | 0.014 | 0.008 | 0.006 | >0.9999 |
| **LPC** | 0.833 | 0.391 | 0.143 | 0.114 | >0.9999 |
| **LPCe** | 0.037 | 0.019 | 0.007 | 0.005 | >0.9999 |
| **LPE** | 0.189 | 0.090 | 0.021 | 0.027 | >0.9999 |
| **LPEp** | 0.140 | 0.110 | 0.027 | 0.035 | >0.9999 |
| **LPS** | 0.005 | 0.003 | 0.001 | 0.001 | >0.9999 |
| **Cer** | 2.048 | 2.203 | 0.480 | 1.560 | >0.9999 |
| **SM** | 9.401 | 3.583 | 1.609 | 1.138 | >0.9999 |
| **LPI** | 0.026 | 0.014 | 0.005 | 0.003 | >0.9999 |
| **CE** | 5.042 | 1.621 | 1.546 | 0.434 | >0.9999 |
| **AC** | 0.790 | 0.733 | 0.041 | 0.074 | >0.9999 |
| **MG** | 3.198 | 2.782 | 0.758 | 0.498 | >0.9999 |
| **DG** | 4.026 | 4.887 | 0.727 | 0.300 | >0.9999 |
| **TG** | 0.140 | 0.122 | 0.028 | 0.011 | >0.9999 |
| **LIPID SPECIES** | | | | | |
| **FREE CHOLESTEROL** | | | | | |
| **FC** | 69.361 | 19.201 | 16.168 | 4.502 | **<0.0001** |
| **PHOSPHATIDYLCHOLINES** | | | | | |
| **PC 30:0** | 21.514 | 8.891 | 1.529 | 2.313 | **<0.0001** |
| **PC 32:0** | 24.376 | 17.060 | 0.927 | 3.063 | **<0.0001** |
| **PC 32:1** | 11.594 | 6.824 | 0.442 | 1.315 | **0.011** |
| **PC 34:0** | 4.063 | 2.330 | 0.160 | 0.608 | >0.9999 |
| **PC 34:1** | 12.054 | 7.337 | 0.295 | 1.587 | **0.013** |
| **PC 34:2** | 8.845 | 5.377 | 0.271 | 1.069 | 0.258 |
| **PC 36:0** | 0.371 | 0.177 | 0.020 | 0.047 | >0.9999 |
| **PC 36:1** | 2.772 | 1.463 | 0.189 | 0.378 | >0.9999 |
| **PC 36:2** | 7.524 | 3.632 | 0.319 | 0.885 | 0.101 |
| **PC 36:3** | 6.271 | 2.965 | 0.261 | 0.729 | 0.361 |
| **PC 36:4** | 7.578 | 2.996 | 0.761 | 0.796 | **0.018** |
| **PC 38:0** | 0.384 | 0.079 | 0.116 | 0.032 | >0.9999 |
| **PC 38:1** | 0.098 | 0.063 | 0.007 | 0.018 | >0.9999 |
| **PC 38:2** | 0.290 | 0.135 | 0.019 | 0.037 | >0.9999 |
| **PC 38:3** | 1.148 | 0.479 | 0.113 | 0.137 | >0.9999 |
| **PC 38:4** | 3.610 | 1.460 | 0.445 | 0.451 | >0.9999 |
| **PC 38:5** | 3.376 | 1.041 | 0.584 | 0.393 | >0.9999 |
| **PC 38:6** | 3.193 | 0.889 | 0.524 | 0.321 | >0.9999 |
| **PC 40:4** | 0.170 | 0.055 | 0.029 | 0.019 | >0.9999 |
| **PC 40:5** | 0.580 | 0.136 | 0.137 | 0.057 | >0.9999 |
| **PC 40:6** | 1.217 | 0.251 | 0.306 | 0.129 | >0.9999 |
| **PC 40:7** | 0.814 | 0.145 | 0.240 | 0.085 | >0.9999 |
| **PC 42:5** | 0.025 | 0.007 | 0.005 | 0.002 | >0.9999 |
| **PC 42:6** | 0.028 | 0.007 | 0.007 | 0.003 | >0.9999 |
| **PC 42:7** | 0.034 | 0.008 | 0.009 | 0.003 | >0.9999 |
| **ETHER PHOSPHATIDYLCHOLINES** | | | | | |
| **PCe 30:0** | 0.955 | 0.227 | 0.201 | 0.060 | **0.009** |
| **PCe 32:0** | 4.638 | 2.046 | 0.263 | 0.527 | **<0.0001** |
| **PCe 32:1** | 0.317 | 0.234 | 0.023 | 0.047 | >0.9999 |
| **PCe 34:0** | 1.426 | 0.852 | 0.047 | 0.198 | >0.9999 |
| **PCe 34:1** | 1.593 | 0.861 | 0.062 | 0.192 | 0.505 |
| **PCe 34:2** | 0.325 | 0.216 | 0.012 | 0.048 | >0.9999 |
| **PCe 36:0** | 0.098 | 0.057 | 0.005 | 0.015 | >0.9999 |
| **PCe 36:1** | 0.596 | 0.348 | 0.032 | 0.091 | >0.9999 |
| **PCe 36:2** | 0.870 | 0.376 | 0.060 | 0.094 | >0.9999 |
| **PCe 36:3** | 0.454 | 0.186 | 0.045 | 0.046 | >0.9999 |
| **PCe 36:4** | 0.699 | 0.328 | 0.085 | 0.095 | >0.9999 |
| **PCe 38:0** | 0.218 | 0.070 | 0.041 | 0.023 | >0.9999 |
| **PCe 38:1** | 0.047 | 0.041 | 0.005 | 0.011 | >0.9999 |
| **PCe 38:2** | 0.098 | 0.068 | 0.011 | 0.019 | >0.9999 |
| **PCe 38:3** | 0.225 | 0.093 | 0.026 | 0.025 | >0.9999 |
| **PCe 38:4** | 0.881 | 0.252 | 0.160 | 0.077 | 0.088 |
| **PCe 38:5** | 1.230 | 0.309 | 0.250 | 0.104 | **0.000** |
| **PCe 38:6** | 0.407 | 0.105 | 0.085 | 0.036 | >0.9999 |
| **PCe 40:4** | 0.105 | 0.031 | 0.023 | 0.009 | >0.9999 |
| **PCe 40:5** | 0.207 | 0.048 | 0.054 | 0.015 | >0.9999 |
| **PCe 40:6** | 0.300 | 0.054 | 0.089 | 0.023 | >0.9999 |
| **PCe 40:7** | 0.371 | 0.077 | 0.112 | 0.032 | >0.9999 |
| **PCe 42:5** | 0.035 | 0.010 | 0.009 | 0.003 | >0.9999 |
| **PCe 42:6** | 0.028 | 0.008 | 0.007 | 0.002 | >0.9999 |
| **PCe 42:7** | 0.021 | 0.008 | 0.004 | 0.002 | >0.9999 |
| **PHOSPHATIDYLETHANOLAMINES** | | | | | |
| **PE 30:0** | 0.062 | 0.025 | 0.018 | 0.007 | >0.9999 |
| **PE 32:0** | 0.077 | 0.045 | 0.026 | 0.021 | >0.9999 |
| **PE 32:1** | 0.143 | 0.027 | 0.060 | 0.013 | >0.9999 |
| **PE 34:0** | 0.114 | 0.040 | 0.023 | 0.012 | >0.9999 |
| **PE 34:1** | 0.716 | 0.166 | 0.196 | 0.059 | **0.005** |
| **PE 34:2** | 0.495 | 0.131 | 0.173 | 0.056 | 0.584 |
| **PE 36:0** | 0.610 | 0.153 | 0.113 | 0.071 | 0.175 |
| **PE 36:1** | 0.367 | 0.129 | 0.102 | 0.045 | >0.9999 |
| **PE 36:2** | 1.091 | 0.208 | 0.409 | 0.092 | **<0.0001** |
| **PE 36:3** | 0.765 | 0.152 | 0.295 | 0.068 | **0.001** |
| **PE 36:4** | 0.406 | 0.101 | 0.150 | 0.038 | >0.9999 |
| **PE 38:0** | 0.538 | 0.162 | 0.117 | 0.050 | 0.535 |
| **PE 38:1** | 0.262 | 0.074 | 0.050 | 0.023 | >0.9999 |
| **PE 38:2** | 0.065 | 0.026 | 0.011 | 0.011 | >0.9999 |
| **PE 38:3** | 0.115 | 0.051 | 0.022 | 0.011 | >0.9999 |
| **PE 38:4** | 0.443 | 0.149 | 0.174 | 0.035 | >0.9999 |
| **PE 38:5** | 0.360 | 0.089 | 0.148 | 0.046 | >0.9999 |
| **PE 38:6** | 0.161 | 0.051 | 0.061 | 0.028 | >0.9999 |
| **PE 40:4** | 0.046 | 0.020 | 0.015 | 0.006 | >0.9999 |
| **PE 40:5** | 0.076 | 0.026 | 0.046 | 0.012 | >0.9999 |
| **PE 40:6** | 0.137 | 0.055 | 0.028 | 0.027 | >0.9999 |
| **PE 40:7** | 0.074 | 0.049 | 0.028 | 0.025 | >0.9999 |
| **PE 42:5** | 0.064 | 0.025 | 0.021 | 0.012 | >0.9999 |
| **PE 42:6** | 0.072 | 0.028 | 0.017 | 0.011 | >0.9999 |
| **PE 42:7** | 0.093 | 0.038 | 0.026 | 0.014 | >0.9999 |
| **PLASMALOGEN PHOSPHATIDYLETHANOLAMINES** | | | | | |
| **PEp 30:0** | 0.010 | 0.021 | 0.003 | 0.007 | >0.9999 |
| **PEp 32:0** | 0.026 | 0.010 | 0.012 | 0.005 | >0.9999 |
| **PEp 32:1** | 0.020 | 0.006 | 0.005 | 0.004 | >0.9999 |
| **PEp 34:0** | 0.065 | 0.030 | 0.024 | 0.011 | >0.9999 |
| **PEp 34:1** | 0.231 | 0.103 | 0.040 | 0.029 | >0.9999 |
| **PEp 34:2** | 0.067 | 0.038 | 0.030 | 0.015 | >0.9999 |
| **PEp 36:0** | 0.092 | 0.031 | 0.038 | 0.012 | >0.9999 |
| **PEp 36:1** | 0.137 | 0.068 | 0.052 | 0.025 | >0.9999 |
| **PEp 36:2** | 0.213 | 0.059 | 0.068 | 0.023 | >0.9999 |
| **PEp 36:3** | 0.177 | 0.053 | 0.046 | 0.017 | >0.9999 |
| **PEp 36:4** | 0.633 | 0.241 | 0.207 | 0.075 | **0.035** |
| **PEp 38:0** | 0.031 | 0.014 | 0.008 | 0.006 | >0.9999 |
| **PEp 38:1** | 0.038 | 0.020 | 0.012 | 0.009 | >0.9999 |
| **PEp 38:2** | 0.046 | 0.022 | 0.011 | 0.009 | >0.9999 |
| **PEp 38:3** | 0.146 | 0.055 | 0.038 | 0.015 | >0.9999 |
| **PEp 38:4** | 0.730 | 0.254 | 0.225 | 0.093 | **0.002** |
| **PEp 38:5** | 0.483 | 0.123 | 0.148 | 0.044 | **0.010** |
| **PEp 38:6** | 0.610 | 0.169 | 0.113 | 0.065 | **0.001** |
| **PEp 40:4** | 0.075 | 0.043 | 0.024 | 0.015 | >0.9999 |
| **PEp 40:5** | 0.121 | 0.050 | 0.048 | 0.019 | >0.9999 |
| **PEp 40:6** | 0.539 | 0.148 | 0.117 | 0.050 | **0.005** |
| **PEp 40:7** | 0.262 | 0.073 | 0.050 | 0.022 | >0.9999 |
| **PEp 42:5** | 0.051 | 0.014 | 0.011 | 0.007 | >0.9999 |
| **PEp 42:6** | 0.068 | 0.040 | 0.021 | 0.014 | >0.9999 |
| **PEp 42:7** | 0.106 | 0.039 | 0.017 | 0.023 | >0.9999 |
| **PHOSPHATIDYLSERINES** | | | | | |
| **PS 30:0** | 0.000 | 0.000 | 0.000 | 0.000 | >0.9999 |
| **PS 32:0** | 0.002 | 0.001 | 0.000 | 0.000 | >0.9999 |
| **PS 32:1** | 0.002 | 0.000 | 0.001 | 0.000 | >0.9999 |
| **PS 34:0** | 0.005 | 0.002 | 0.001 | 0.001 | >0.9999 |
| **PS 34:1** | 0.033 | 0.007 | 0.009 | 0.003 | >0.9999 |
| **PS 34:2** | 0.016 | 0.002 | 0.004 | 0.001 | >0.9999 |
| **PS 36:0** | 0.015 | 0.004 | 0.005 | 0.001 | >0.9999 |
| **PS 36:1** | 0.140 | 0.031 | 0.040 | 0.011 | **<0.0001** |
| **PS 36:2** | 0.113 | 0.017 | 0.045 | 0.008 | **<0.0001** |
| **PS 36:3** | 0.025 | 0.003 | 0.011 | 0.002 | >0.9999 |
| **PS 36:4** | 0.010 | 0.001 | 0.004 | 0.001 | >0.9999 |
| **PS 38:0** | 0.001 | 0.000 | 0.001 | 0.000 | >0.9999 |
| **PS 38:1** | 0.010 | 0.002 | 0.003 | 0.001 | >0.9999 |
| **PS 38:2** | 0.014 | 0.002 | 0.005 | 0.001 | >0.9999 |
| **PS 38:3** | 0.029 | 0.005 | 0.010 | 0.002 | >0.9999 |
| **PS 38:4** | 0.081 | 0.012 | 0.031 | 0.007 | **<0.0001** |
| **PS 38:5** | 0.019 | 0.002 | 0.008 | 0.001 | >0.9999 |
| **PS 38:6** | 0.002 | 0.000 | 0.001 | 0.000 | >0.9999 |
| **PS 40:4** | 0.010 | 0.002 | 0.003 | 0.001 | >0.9999 |
| **PS 40:5** | 0.012 | 0.002 | 0.004 | 0.001 | >0.9999 |
| **PS 40:6** | 0.013 | 0.002 | 0.004 | 0.001 | >0.9999 |
| **PS 40:7** | 0.002 | 0.000 | 0.001 | 0.000 | >0.9999 |
| **PS 42:5** | 0.002 | 0.000 | 0.001 | 0.000 | >0.9999 |
| **PS 42:6** | 0.001 | 0.000 | 0.000 | 0.000 | >0.9999 |
| **PS 42:7** | 0.000 | 0.000 | 0.000 | 0.000 | >0.9999 |
| **PHOSPHATIDYLINOSITOLS** | | | | | |
| **PI 30:0** | 0.007 | 0.002 | 0.002 | 0.001 | >0.9999 |
| **PI 32:0** | 0.038 | 0.005 | 0.017 | 0.002 | >0.9999 |
| **PI 32:1** | 0.021 | 0.003 | 0.008 | 0.001 | >0.9999 |
| **PI 34:0** | 0.071 | 0.010 | 0.027 | 0.004 | >0.9999 |
| **PI 34:1** | 0.387 | 0.068 | 0.101 | 0.026 | **<0.0001** |
| **PI 34:2** | 0.085 | 0.019 | 0.020 | 0.006 | >0.9999 |
| **PI 36:0** | 0.045 | 0.010 | 0.011 | 0.004 | >0.9999 |
| **PI 36:1** | 0.444 | 0.091 | 0.108 | 0.036 | **<0.0001** |
| **PI 36:2** | 0.467 | 0.096 | 0.113 | 0.039 | **<0.0001** |
| **PI 36:3** | 0.099 | 0.024 | 0.025 | 0.010 | >0.9999 |
| **PI 36:4** | 0.040 | 0.011 | 0.010 | 0.004 | >0.9999 |
| **PI 38:0** | 0.003 | 0.001 | 0.001 | 0.000 | >0.9999 |
| **PI 38:1** | 0.005 | 0.002 | 0.002 | 0.001 | >0.9999 |
| **PI 38:2** | 0.018 | 0.004 | 0.004 | 0.001 | >0.9999 |
| **PI 38:3** | 0.047 | 0.012 | 0.011 | 0.004 | >0.9999 |
| **PI 38:4** | 0.152 | 0.040 | 0.036 | 0.012 | 0.327 |
| **PI 38:5** | 0.080 | 0.015 | 0.021 | 0.006 | >0.9999 |
| **PI 38:6** | 0.049 | 0.009 | 0.016 | 0.004 | >0.9999 |
| **PI 40:4** | 0.010 | 0.003 | 0.004 | 0.001 | >0.9999 |
| **PI 40:5** | 0.027 | 0.005 | 0.010 | 0.003 | >0.9999 |
| **PI 40:6** | 0.030 | 0.006 | 0.008 | 0.003 | >0.9999 |
| **PI 40:7** | 0.010 | 0.002 | 0.003 | 0.001 | >0.9999 |
| **PI 42:5** | 0.001 | 0.000 | 0.000 | 0.000 | >0.9999 |
| **PI 42:6** | 0.002 | 0.001 | 0.001 | 0.000 | >0.9999 |
| **PI 42:7** | 0.002 | 0.001 | 0.000 | 0.000 | >0.9999 |
| **PHOSPHATIDYLGLYCEROLS** | | | | | |
| **PG 30:0** | 0.172 | 0.035 | 0.057 | 0.017 | >0.9999 |
| **PG 32:0** | 0.041 | 0.011 | 0.009 | 0.006 | >0.9999 |
| **PG 32:1** | 0.536 | 0.149 | 0.088 | 0.066 | >0.9999 |
| **PG 34:0** | 0.522 | 0.201 | 0.052 | 0.086 | >0.9999 |
| **PG 34:1** | 3.379 | 1.326 | 0.209 | 0.551 | **0.001** |
| **PG 34:2** | 0.452 | 0.201 | 0.036 | 0.072 | >0.9999 |
| **PG 36:0** | 0.185 | 0.080 | 0.014 | 0.031 | >0.9999 |
| **PG 36:1** | 1.458 | 0.615 | 0.128 | 0.238 | >0.9999 |
| **PG 36:2** | 6.214 | 2.188 | 0.401 | 0.782 | **<0.0001** |
| **PG 36:3** | 0.036 | 0.012 | 0.005 | 0.005 | >0.9999 |
| **PG 36:4** | 0.280 | 0.078 | 0.056 | 0.037 | >0.9999 |
| **PG 38:0** | 0.003 | 0.002 | 0.001 | 0.001 | >0.9999 |
| **PG 38:1** | 0.022 | 0.008 | 0.004 | 0.003 | >0.9999 |
| **PG 38:2** | 0.041 | 0.012 | 0.007 | 0.005 | >0.9999 |
| **PG 38:3** | 0.029 | 0.008 | 0.004 | 0.004 | >0.9999 |
| **PG 38:4** | 0.307 | 0.085 | 0.080 | 0.050 | >0.9999 |
| **PG 38:5** | 0.172 | 0.036 | 0.046 | 0.018 | >0.9999 |
| **PG 38:6** | 0.199 | 0.038 | 0.052 | 0.021 | >0.9999 |
| **PG 40:4** | 0.012 | 0.003 | 0.004 | 0.002 | >0.9999 |
| **PG 40:5** | 0.048 | 0.011 | 0.017 | 0.006 | >0.9999 |
| **PG 40:6** | 0.187 | 0.035 | 0.057 | 0.022 | >0.9999 |
| **PG 40:7** | 0.096 | 0.021 | 0.027 | 0.012 | >0.9999 |
| **PG 42:5** | 0.008 | 0.003 | 0.001 | 0.001 | >0.9999 |
| **PG 42:6** | 0.019 | 0.009 | 0.004 | 0.003 | >0.9999 |
| **PG 42:7** | 0.019 | 0.008 | 0.005 | 0.002 | >0.9999 |
| **PHOSPHATIDIC ACIDS** | | | | | |
| **PA 30:0** | 0.009 | 0.007 | 0.001 | 0.001 | >0.9999 |
| **PA 32:0** | 0.009 | 0.006 | 0.001 | 0.002 | >0.9999 |
| **PA 32:1** | 0.006 | 0.005 | 0.001 | 0.001 | >0.9999 |
| **PA 34:0** | 0.006 | 0.006 | 0.001 | 0.002 | >0.9999 |
| **PA 34:1** | 0.019 | 0.010 | 0.002 | 0.003 | >0.9999 |
| **PA 34:2** | 0.011 | 0.008 | 0.001 | 0.002 | >0.9999 |
| **PA 36:0** | 0.005 | 0.003 | 0.001 | 0.001 | >0.9999 |
| **PA 36:1** | 0.036 | 0.022 | 0.006 | 0.004 | >0.9999 |
| **PA 36:2** | 0.039 | 0.015 | 0.004 | 0.003 | <0.0001 |
| **PA 36:3** | 0.015 | 0.008 | 0.001 | 0.002 | >0.9999 |
| **PA 36:4** | 0.010 | 0.007 | 0.001 | 0.001 | >0.9999 |
| **PA 38:0** | 0.006 | 0.004 | 0.001 | 0.001 | >0.9999 |
| **PA 38:1** | 0.009 | 0.005 | 0.001 | 0.001 | >0.9999 |
| **PA 38:2** | 0.009 | 0.005 | 0.001 | 0.001 | >0.9999 |
| **PA 38:3** | 0.013 | 0.007 | 0.003 | 0.001 | >0.9999 |
| **PA 38:4** | 0.028 | 0.013 | 0.003 | 0.003 | 0.147 |
| **PA 38:5** | 0.022 | 0.010 | 0.004 | 0.002 | 0.524 |
| **PA 38:6** | 0.008 | 0.004 | 0.001 | 0.001 | >0.9999 |
| **PA 40:4** | 0.006 | 0.004 | 0.001 | 0.001 | >0.9999 |
| **PA 40:5** | 0.012 | 0.005 | 0.002 | 0.001 | >0.9999 |
| **PA 40:6** | 0.030 | 0.013 | 0.009 | 0.003 | 0.087 |
| **PA 40:7** | 0.010 | 0.005 | 0.002 | 0.001 | >0.9999 |
| **PA 42:5** | 0.007 | 0.005 | 0.001 | 0.001 | >0.9999 |
| **PA 42:6** | 0.033 | 0.013 | 0.009 | 0.003 | **0.004** |
| **PA 42:7** | 0.026 | 0.006 | 0.008 | 0.002 | **0.002** |
| **BMP** | | | | | |
| **BMP 30:0** | 0.002 | 0.001 | 0.001 | 0.000 | >0.9999 |
| **BMP 32:0** | 0.003 | 0.001 | 0.001 | 0.001 | >0.9999 |
| **BMP 32:1** | 0.009 | 0.003 | 0.002 | 0.001 | >0.9999 |
| **BMP 34:0** | 0.119 | 0.038 | 0.026 | 0.019 | >0.9999 |
| **BMP 34:1** | 0.306 | 0.095 | 0.039 | 0.043 | 0.928 |
| **BMP 34:2** | 0.056 | 0.023 | 0.007 | 0.011 | >0.9999 |
| **BMP 36:0** | 0.233 | 0.088 | 0.054 | 0.048 | >0.9999 |
| **BMP 36:1** | 0.762 | 0.244 | 0.127 | 0.119 | **<0.0001** |
| **BMP 36:2** | 1.680 | 0.528 | 0.180 | 0.231 | **<0.0001** |
| **BMP 36:3** | 0.005 | 0.002 | 0.001 | 0.001 | >0.9999 |
| **BMP 36:4** | 0.009 | 0.004 | 0.001 | 0.002 | >0.9999 |
| **BMP 38:0** | 0.002 | 0.001 | 0.001 | 0.000 | >0.9999 |
| **BMP 38:1** | 0.007 | 0.003 | 0.002 | 0.001 | >0.9999 |
| **BMP 38:2** | 0.015 | 0.005 | 0.002 | 0.002 | >0.9999 |
| **BMP 38:3** | 0.012 | 0.004 | 0.001 | 0.002 | >0.9999 |
| **BMP 38:4** | 0.022 | 0.008 | 0.004 | 0.004 | >0.9999 |
| **BMP 38:5** | 0.010 | 0.004 | 0.001 | 0.002 | >0.9999 |
| **BMP 38:6** | 0.022 | 0.007 | 0.007 | 0.004 | >0.9999 |
| **BMP 40:4** | 0.008 | 0.003 | 0.001 | 0.001 | >0.9999 |
| **BMP 40:5** | 0.031 | 0.011 | 0.007 | 0.006 | >0.9999 |
| **BMP 40:6** | 0.089 | 0.024 | 0.031 | 0.015 | >0.9999 |
| **BMP 40:7** | 0.066 | 0.021 | 0.018 | 0.010 | >0.9999 |
| **BMP 42:5** | 0.006 | 0.002 | 0.000 | 0.001 | >0.9999 |
| **BMP 42:6** | 0.017 | 0.005 | 0.000 | 0.002 | >0.9999 |
| **BMP 42:7** | 0.015 | 0.005 | 0.002 | 0.002 | >0.9999 |
| **ACYL PHOSPHATIDYLGLYCEROLS** | | | | | |
| **AcylPG 16:0-34:0** | 0.002 | 0.001 | 0.001 | 0.000 | >0.9999 |
| **AcylPG 16:0-34:1** | 0.009 | 0.003 | 0.002 | 0.001 | **0.005** |
| **AcylPG 16:0-34:2** | 0.002 | 0.001 | 0.000 | 0.000 | >0.9999 |
| **AcylPG 16:0-36:0** | 0.001 | 0.000 | 0.000 | 0.000 | >0.9999 |
| **AcylPG 16:0-36:1** | 0.006 | 0.001 | 0.001 | 0.000 | **0.027** |
| **AcylPG 16:0-36:2** | 0.011 | 0.003 | 0.003 | 0.001 | <0.0001 |
| **AcylPG 16:0-36:3** | 0.003 | 0.001 | 0.001 | 0.000 | >0.9999 |
| **AcylPG 16:0-36:4** | 0.001 | 0.000 | 0.000 | 0.000 | >0.9999 |
| **AcylPG 16:0-38:1** | 0.001 | 0.000 | 0.000 | 0.000 | >0.9999 |
| **AcylPG 16:0-38:2** | 0.004 | 0.001 | 0.000 | 0.001 | >0.9999 |
| **AcylPG 16:0-38:3** | 0.005 | 0.001 | 0.001 | 0.001 | 0.316 |
| **AcylPG 16:0-38:4** | 0.001 | 0.000 | 0.000 | 0.000 | >0.9999 |
| **AcylPG 16:0-38:5** | 0.001 | 0.000 | 0.000 | 0.000 | >0.9999 |
| **AcylPG 16:0-40:5** | 0.000 | 0.000 | 0.000 | 0.000 | >0.9999 |
| **AcylPG 16:0-40:6** | 0.002 | 0.000 | 0.000 | 0.000 | >0.9999 |
| **LYSOPHOSPHOLIPIDS** | | | | | |
| **LPC 16:0** | 0.458 | 0.227 | 0.080 | 0.069 | <0.0001 |
| **LPC 16:1** | 0.070 | 0.032 | 0.010 | 0.009 | >0.9999 |
| **LPC 18:0** | 0.108 | 0.049 | 0.027 | 0.015 | 0.8752 |
| **LPC 18:1** | 0.157 | 0.062 | 0.025 | 0.017 | 0.068 |
| **LPC 20:0** | 0.003 | 0.001 | 0.001 | 0.000 | >0.9999 |
| **LPC 20:1** | 0.002 | 0.001 | 0.000 | 0.000 | >0.9999 |
| **LPC 20:2** | 0.003 | 0.001 | 0.001 | 0.000 | >0.9999 |
| **LPC 20:3** | 0.007 | 0.003 | 0.001 | 0.001 | >0.9999 |
| **LPC 20:4** | 0.025 | 0.014 | 0.005 | 0.006 | >0.9999 |
| **LPCe 16:0** | 0.007 | 0.004 | 0.001 | 0.001 | >0.9999 |
| **LPCe 16:1** | 0.002 | 0.001 | 0.000 | 0.000 | >0.9999 |
| **LPCe 18:0** | 0.022 | 0.010 | 0.005 | 0.003 | <0.0001 |
| **LPCe 18:1** | 0.005 | 0.002 | 0.001 | 0.001 | >0.9999 |
| **LPCe 20:0** | 0.001 | 0.001 | 0.000 | 0.000 | >0.9999 |
| **LPCe 20:1** | 0.001 | 0.001 | 0.000 | 0.000 | >0.9999 |
| **LPCe 20:2** | 0.000 | 0.000 | 0.000 | 0.000 | >0.9999 |
| **LPE 16:0** | 0.047 | 0.024 | 0.009 | 0.007 | >0.9999 |
| **LPE 18:0** | 0.052 | 0.029 | 0.010 | 0.010 | >0.9999 |
| **LPE 18:1** | 0.090 | 0.037 | 0.019 | 0.013 | **0.0038** |
| **LPEp 16:0** | 0.028 | 0.044 | 0.022 | 0.012 | 0.4908 |
| **LPEp 18:0** | 0.076 | 0.046 | 0.012 | 0.018 | >0.9999 |
| **LPEp 18:1** | 0.028 | 0.016 | 0.011 | 0.005 | >0.9999 |
| **LPEp 20:0** | 0.007 | 0.005 | 0.002 | 0.002 | >0.9999 |
| **LPI 16:0** | 0.003 | 0.002 | 0.000 | 0.000 | >0.9999 |
| **LPI 16:1** | 0.000 | 0.000 | 0.000 | 0.000 | >0.9999 |
| **LPI 18:0** | 0.014 | 0.007 | 0.004 | 0.001 | **0.0003** |
| **LPI 18:1** | 0.006 | 0.003 | 0.001 | 0.001 | 0.0917 |
| **LPI 20:3** | 0.000 | 0.000 | 0.000 | 0.000 | >0.9999 |
| **LPI 20:4** | 0.001 | 0.001 | 0.000 | 0.000 | >0.9999 |
| **LPS 16:0** | 0.000 | 0.000 | 0.000 | 0.000 | >0.9999 |
| **LPS 18:0** | 0.002 | 0.002 | 0.000 | 0.000 | 0.0601 |
| **LPS 18:1** | 0.002 | 0.001 | 0.000 | 0.000 | **0.0352** |
| **LPS 20:4** | 0.000 | 0.000 | 0.000 | 0.000 | >0.9999 |
| **LPS 22:0** | 0.000 | 0.000 | 0.000 | 0.000 | >0.9999 |
| **LPS 22:1** | 0.000 | 0.000 | 0.000 | 0.000 | >0.9999 |
| **LPS 24:0** | 0.000 | 0.000 | 0.000 | 0.000 | >0.9999 |
| **LPS 24:1** | 0.000 | 0.000 | 0.000 | 0.000 | >0.9999 |
| **CERAMIDES** | | | | | |
| **Cer d18:1/16:0** | 0.224 | 0.178 | 0.048 | 0.102 | >0.9999 |
| **Cer d18:1/16:1** | 0.276 | 0.219 | 0.060 | 0.043 | >0.9999 |
| **Cer d18:1/18:0** | 0.169 | 0.289 | 0.031 | 0.260 | >0.9999 |
| **Cer d18:1/18:1** | 0.187 | 0.313 | 0.124 | 0.256 | >0.9999 |
| **Cer d18:1/20:0** | 0.124 | 0.266 | 0.056 | 0.241 | >0.9999 |
| **Cer d18:1/20:1** | 0.207 | 0.304 | 0.064 | 0.256 | >0.9999 |
| **Cer d18:1/22:0** | 0.128 | 0.182 | 0.059 | 0.160 | >0.9999 |
| **Cer d18:1/22:1** | 0.173 | 0.079 | 0.042 | 0.027 | >0.9999 |
| **Cer d18:1/24:0** | 0.205 | 0.131 | 0.080 | 0.073 | >0.9999 |
| **Cer d18:1/24:1** | 0.174 | 0.049 | 0.071 | 0.014 | >0.9999 |
| **Cer d18:1/26:0** | 0.152 | 0.178 | 0.064 | 0.163 | >0.9999 |
| **Cer d18:1/26:1** | 0.029 | 0.017 | 0.006 | 0.006 | >0.9999 |
| **SPHINGOMYELINS** | | | | | |
| **SM d18:1/16:0** | 5.389 | 2.088 | 0.950 | 0.700 | **<0.0001** |
| **SM d18:1/16:1** | 0.164 | 0.059 | 0.038 | 0.018 | >0.9999 |
| **SM d18:1/18:0** | 0.225 | 0.107 | 0.050 | 0.027 | >0.9999 |
| **SM d18:1/18:1** | 0.029 | 0.016 | 0.008 | 0.005 | >0.9999 |
| **SM d18:1/20:0** | 0.367 | 0.138 | 0.057 | 0.039 | >0.9999 |
| **SM d18:1/20:1** | 0.035 | 0.016 | 0.008 | 0.005 | >0.9999 |
| **SM d18:1/22:0** | 0.861 | 0.328 | 0.154 | 0.105 | >0.9999 |
| **SM d18:1/22:1** | 0.114 | 0.054 | 0.020 | 0.016 | >0.9999 |
| **SM d18:1/24:0** | 1.247 | 0.455 | 0.223 | 0.162 | >0.9999 |
| **SM d18:1/24:1** | 0.910 | 0.306 | 0.157 | 0.097 | >0.9999 |
| **SM d18:1/26:0** | 0.032 | 0.009 | 0.004 | 0.004 | >0.9999 |
| **SM d18:1/26:1** | 0.028 | 0.008 | 0.003 | 0.003 | >0.9999 |
| **CHOLESTEROL ESTERS** | | | | | |
| **CE 16:0** | 0.354 | 0.117 | 0.193 | 0.050 | >0.9999 |
| **CE 16:1** | 0.272 | 0.089 | 0.165 | 0.058 | >0.9999 |
| **CE 18:0** | 0.055 | 0.023 | 0.029 | 0.007 | >0.9999 |
| **CE 18:1** | 1.116 | 0.445 | 0.596 | 0.277 | >0.9999 |
| **CE 18:2** | 2.157 | 2.510 | 0.501 | 1.729 | >0.9999 |
| **CE 20:1** | 0.024 | 0.006 | 0.016 | 0.001 | >0.9999 |
| **CE 20:2** | 0.065 | 0.011 | 0.039 | 0.003 | >0.9999 |
| **CE 20:3** | 0.176 | 0.088 | 0.070 | 0.060 | >0.9999 |
| **CE 20:4** | 0.587 | 0.561 | 0.160 | 0.364 | >0.9999 |
| **CE 22:3** | 0.011 | 0.004 | 0.005 | 0.001 | >0.9999 |
| **CE 22:4** | 0.055 | 0.007 | 0.035 | 0.002 | >0.9999 |
| **CE 22:5** | 0.063 | 0.014 | 0.039 | 0.005 | >0.9999 |
| **CE 22:6** | 0.098 | 0.061 | 0.044 | 0.045 | >0.9999 |
| **CE 24:4** | 0.009 | 0.002 | 0.004 | 0.000 | >0.9999 |
| **ACYL CARNITINES** | | | | | |
| **AC C12:0** | 0.017 | 0.018 | 0.003 | 0.009 | >0.9999 |
| **AC C14:0** | 0.052 | 0.053 | 0.005 | 0.007 | >0.9999 |
| **AC C16:0** | 0.435 | 0.437 | 0.030 | 0.044 | >0.9999 |
| **AC C18:0** | 0.143 | 0.134 | 0.012 | 0.013 | >0.9999 |
| **AC C18:1** | 0.099 | 0.065 | 0.009 | 0.006 | >0.9999 |
| **AC C2:0** | 0.005 | 0.002 | 0.001 | 0.000 | >0.9999 |
| **AC C3:0** | 0.002 | 0.001 | 0.000 | 0.000 | >0.9999 |
| **AC C6:0** | 0.007 | 0.002 | 0.003 | 0.000 | >0.9999 |
| **AC C8:0** | 0.030 | 0.020 | 0.005 | 0.003 | >0.9999 |
| **MONOACYLGLYCEROLS** | | | | | |
| **MG 16:0** | 0.320 | 0.214 | 0.086 | 0.066 | >0.9999 |
| **MG 16:1** | 0.139 | 0.274 | 0.027 | 0.067 | >0.9999 |
| **MG 18:0** | 0.514 | 0.225 | 0.122 | 0.058 | >0.9999 |
| **MG 18:1** | 0.106 | 0.113 | 0.011 | 0.023 | >0.9999 |
| **MG 18:2** | 0.101 | 0.162 | 0.020 | 0.026 | >0.9999 |
| **MG 18:3** | 0.098 | 0.109 | 0.012 | 0.027 | >0.9999 |
| **MG 20:0** | 0.393 | 0.116 | 0.288 | 0.026 | >0.9999 |
| **MG 20:1** | 0.315 | 0.313 | 0.026 | 0.121 | >0.9999 |
| **MG 20:2** | 0.085 | 0.097 | 0.014 | 0.025 | >0.9999 |
| **MG 20:3** | 0.132 | 0.112 | 0.057 | 0.039 | >0.9999 |
| **MG 20:4** | 0.088 | 0.111 | 0.012 | 0.030 | >0.9999 |
| **MG 22:0** | 0.390 | 0.276 | 0.143 | 0.099 | >0.9999 |
| **MG 22:1** | 0.073 | 0.110 | 0.011 | 0.032 | >0.9999 |
| **MG 22:2** | 0.083 | 0.076 | 0.026 | 0.025 | >0.9999 |
| **MG 22:3** | 0.106 | 0.054 | 0.060 | 0.015 | >0.9999 |
| **MG 22:4** | 0.102 | 0.051 | 0.026 | 0.012 | >0.9999 |
| **MG 22:5** | 0.060 | 0.284 | 0.017 | 0.111 | >0.9999 |
| **MG 22:6** | 0.091 | 0.083 | 0.024 | 0.023 | >0.9999 |
| **DIACYLGLYCEROLS** | | | | | |
| **DG 28:0/14:0** | 2.891 | 4.223 | 0.571 | 0.293 | >0.9999 |
| **DG 30:0/14:0** | 0.013 | 0.010 | 0.003 | 0.001 | >0.9999 |
| **DG 30:1/14:0** | 0.004 | 0.003 | 0.001 | 0.000 | >0.9999 |
| **DG 32:0/16:0** | 0.064 | 0.020 | 0.017 | 0.004 | >0.9999 |
| **DG 32:1/16:0** | 0.026 | 0.014 | 0.004 | 0.002 | >0.9999 |
| **DG 32:2/16:1** | 0.011 | 0.012 | 0.002 | 0.003 | >0.9999 |
| **DG 34:0/16:0** | 0.055 | 0.050 | 0.012 | 0.005 | >0.9999 |
| **DG 34:1/16:0** | 0.099 | 0.029 | 0.017 | 0.006 | >0.9999 |
| **DG 34:2/16:0** | 0.059 | 0.031 | 0.006 | 0.006 | >0.9999 |
| **DG 34:2/16:1** | 0.028 | 0.018 | 0.005 | 0.005 | >0.9999 |
| **DG 36:0/18:0** | 0.245 | 0.251 | 0.060 | 0.032 | >0.9999 |
| **DG 36:1/18:0** | 0.124 | 0.044 | 0.021 | 0.012 | >0.9999 |
| **DG 36:2/18:0** | 0.023 | 0.011 | 0.003 | 0.002 | >0.9999 |
| **DG 36:2/18:1** | 0.243 | 0.077 | 0.054 | 0.019 | >0.9999 |
| **DG 36:3/18:1** | 0.079 | 0.040 | 0.017 | 0.009 | >0.9999 |
| **DG 38:2/18:0** | 0.005 | 0.004 | 0.001 | 0.001 | >0.9999 |
| **DG 38:2/18:1** | 0.005 | 0.003 | 0.001 | 0.001 | >0.9999 |
| **DG 38:3/18:0** | 0.022 | 0.025 | 0.002 | 0.002 | >0.9999 |
| **DG 38:3/18:1** | 0.003 | 0.002 | 0.000 | 0.000 | >0.9999 |
| **DG 38:4/18:0** | 0.007 | 0.004 | 0.001 | 0.001 | >0.9999 |
| **DG 38:4/18:1** | 0.007 | 0.006 | 0.001 | 0.001 | >0.9999 |
| **DG 40:4/18:0** | 0.003 | 0.002 | 0.001 | 0.000 | >0.9999 |
| **DG 40:4/18:1** | 0.001 | 0.001 | 0.000 | 0.000 | >0.9999 |
| **DG 40:5/18:0** | 0.003 | 0.002 | 0.000 | 0.000 | >0.9999 |
| **DG 40:5/18:1** | 0.001 | 0.001 | 0.000 | 0.000 | >0.9999 |
| **DG 40:6/18:0** | 0.004 | 0.002 | 0.000 | 0.000 | >0.9999 |
| **DG 40:6/18:1** | 0.002 | 0.001 | 0.000 | 0.000 | >0.9999 |
| **TRIACYLGLYCEROLS** | | | | | |
| **TG 48:0/16:0** | 0.028 | 0.023 | 0.013 | 0.006 | >0.9999 |
| **TG 48:1/16:0** | 0.014 | 0.025 | 0.004 | 0.009 | >0.9999 |
| **TG 50:0/16:0** | 0.009 | 0.012 | 0.003 | 0.004 | >0.9999 |
| **TG 50:1/16:1** | 0.003 | 0.005 | 0.000 | 0.002 | >0.9999 |
| **TG 50:2/16:1** | 0.007 | 0.022 | 0.001 | 0.013 | >0.9999 |
| **TG 50:3/16:1** | 0.008 | 0.030 | 0.001 | 0.016 | >0.9999 |
| **TG 52:0/18:0** | 0.005 | 0.006 | 0.001 | 0.001 | >0.9999 |
| **TG 52:1/18:0** | 0.008 | 0.010 | 0.001 | 0.005 | >0.9999 |
| **TG 52:2/18:0** | 0.003 | 0.006 | 0.001 | 0.003 | >0.9999 |
| **TG 52:3/18:1** | 0.015 | 0.063 | 0.003 | 0.040 | >0.9999 |
| **TG 52:4/18:1** | 0.002 | 0.012 | 0.000 | 0.006 | >0.9999 |
| **TG 52:5/18:1** | 0.000 | 0.001 | 0.000 | 0.001 | >0.9999 |
| **TG 52:5/20:4** | 0.000 | 0.001 | 0.000 | 0.001 | >0.9999 |
| **TG 54:0/18:0** | 0.007 | 0.012 | 0.001 | 0.002 | >0.9999 |
| **TG 54:1/18:0** | 0.002 | 0.002 | 0.000 | 0.000 | >0.9999 |
| **TG 54:2/18:0** | 0.004 | 0.005 | 0.001 | 0.002 | >0.9999 |
| **TG 54:3/18:0** | 0.003 | 0.005 | 0.000 | 0.002 | >0.9999 |
| **TG 54:4/18:1** | 0.009 | 0.024 | 0.002 | 0.012 | >0.9999 |
| **TG 54:4/20:4** | 0.001 | 0.001 | 0.000 | 0.000 | >0.9999 |
| **TG 54:5/18:1** | 0.004 | 0.008 | 0.001 | 0.003 | >0.9999 |
| **TG 54:5/20:4** | 0.001 | 0.003 | 0.000 | 0.002 | >0.9999 |
| **TG 54:6/18:1** | 0.001 | 0.002 | 0.000 | 0.001 | >0.9999 |
| **TG 54:6/20:4** | 0.001 | 0.002 | 0.000 | 0.001 | >0.9999 |
| **TG 54:7/18:1** | 0.000 | 0.000 | 0.000 | 0.000 | >0.9999 |
| **TG 54:7/20:4** | 0.000 | 0.000 | 0.000 | 0.000 | >0.9999 |
| **TG 56:3/18:1** | 0.001 | 0.001 | 0.000 | 0.000 | >0.9999 |
| **TG 56:4/18:1** | 0.001 | 0.001 | 0.000 | 0.001 | >0.9999 |
| **TG 56:4/20:4** | 0.000 | 0.000 | 0.000 | 0.000 | >0.9999 |
| **TG 56:5/18:1** | 0.001 | 0.001 | 0.000 | 0.001 | >0.9999 |
| **TG 56:5/20:4** | 0.001 | 0.001 | 0.000 | 0.001 | >0.9999 |
| **TG 56:6/20:4** | 0.000 | 0.002 | 0.000 | 0.001 | >0.9999 |
| **TG 56:7/20:4** | 0.000 | 0.001 | 0.000 | 0.001 | >0.9999 |
| **TG 56:8/20:4** | 0.000 | 0.000 | 0.000 | 0.000 | >0.9999 |
| **TG 58:8/22:6** | 0.000 | 0.001 | 0.000 | 0.000 | >0.9999 |
| **TG 58:9/22:6** | 0.000 | 0.000 | 0.000 | 0.000 | >0.9999 |
